# Manipulating Phospholipid Vesicles at the Nanoscale: A Transformation from Unilamellar to Multilamellar by an *n*-Alkyl-poly(ethylene oxide)

**DOI:** 10.1021/acs.langmuir.0c03302

**Published:** 2021-02-11

**Authors:** Judith U. De Mel, Sudipta Gupta, Lutz Willner, Jürgen Allgaier, Laura R. Stingaciu, Markus Bleuel, Gerald J. Schneider

**Affiliations:** ^†^Department of Chemistry and ^⊥^Department of Physics & Astronomy, Louisiana State University, Baton Rouge, Louisiana 70803, United States; ‡ Jülich Center for Neutron Science (JCNS-1) and Institute of Biological Information Processing (IBI-8) Forschungszentrum Jülich GmbH, 52428 Jülich, Germany; § Neutron Sciences Directorate, Oak Ridge National Laboratory (ORNL), POB 2008, 1 Bethel Valley Road, Oak Ridge, Tennessee 37831, United States; ∥ NIST Center for Neutron Research, National Institute of Standards and Technology, Gaithersburg, Maryland 20899-8562, United States

## Abstract

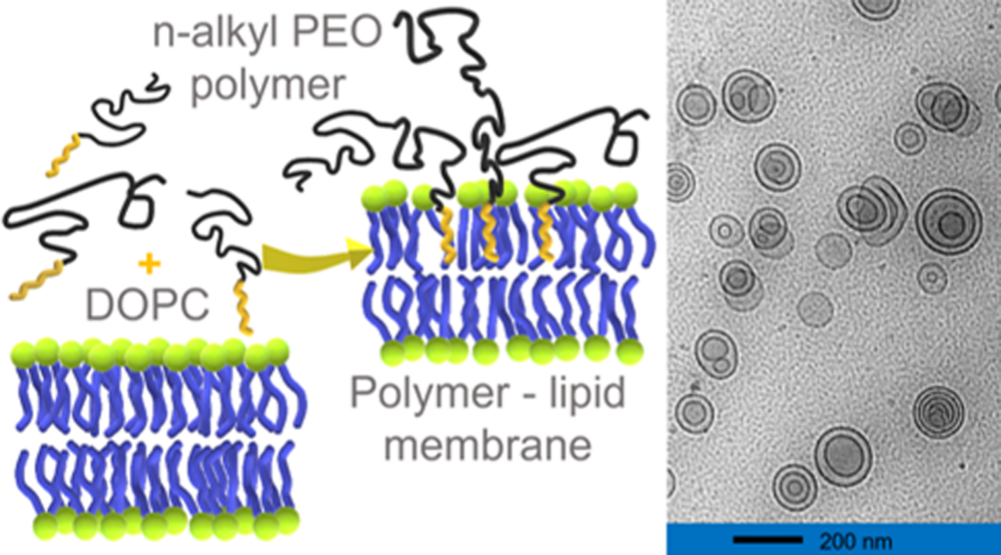

We investigated the influence of an *n*-alkyl-PEO polymer on the structure and dynamics of phospholipid vesicles. Multilayer formation and about a 9% increase in the size in vesicles were observed by cryogenic transmission electron microscopy (cryo-TEM), dynamic light scattering (DLS), and small-angle neutron/X-ray scattering (SANS/SAXS). The results indicate a change in the lamellar structure of the vesicles by a partial disruption caused by polymer chains, which seems to correlate with about a 30% reduction in bending rigidity per unit bilayer, as revealed by neutron spin echo (NSE) spectroscopy. Also, a strong change in lipid tail relaxation was observed. Our results point to opportunities using synthetic polymers to control the structure and dynamics of membranes, with possible applications in technical materials and also in drug and nutraceutical delivery.

## Introduction

Phospholipid vesicles are a versatile system for a variety of applications.^1−3^ Primarily made up of phospholipids that have a polar headgroup and hydrophobic tails, these are spherical self-assemblies that can span sizes from tens of nanometers to several micrometers. Despite the size differences, the structure is universal for vesicles with an aqueous core encapsulated by a phospholipid bilayer with the polar headgroups of the two layers facing opposite directions, allowing the lipid tails to be sandwiched in the middle. The properties of phospholipid vesicles can be changed using additives such as cholesterol, small molecules, and macromolecules such as synthetic polymers.^4−12^ From the initial stages of incorporating synthetic polymers into phospholipid vesicles, the present vesicle formulations with associated polymers have come a long way in terms of fine tuning their properties for drug delivery and applications in cosmetics, nutraceuticals, and food technology.^13^ Polymer–liposome interactions could lead to (i) coating, (ii) insertion, (iii) disruption, and/or (iv) transforming the entire vesicle self-assembly.^14^ These interactions are susceptible to experimental parameters such as solvent quality, temperature, ionic strength, and pressure and ultimately determine the liposome morphology, bilayer structure, and dynamics in the presence of polymers. Altogether, they can unfold a plethora of benefits in applications when it comes to the (i) encapsulation efficiency, (ii) controlled release, and (iii) drug transport mechanisms such as skin penetration.^15,16^ Despite the abundance of applications pointing to the importance of molecular-level interactions, fundamental questions such as how polymer–liposome interactions on the nanoscale drive the structure and dynamic changes of these self-assemblies are not well understood.

One popular polymer for studying interactions with phospholipid membranes has been PEG (poly(ethylene glycol)), which has a repeating unit identical to that of PEO (poly(ethylene oxide)) or POE (polyoxyethylene), −(CH_2_–CH_2_–O−)_*n*_. Throughout the article, we will use the abbreviation PEO to be consistent. There are a variety of applications of PEOs from steric stabilization agents^17^ to formulated “stealth” vesicles which increase blood circulation times.^18^ Although the biocompatibility of PEO is well established, higher-molecular-weight PEOs are known to show various levels of cytotoxicity and aggregation.^19,20^ Therefore, we decided on a PEO-based polymer with a low degree of polymerization (*n* < 100) for this work. PEO chains when functionalized with hydrophobic molecules, such as pairs of dodecyl or cholesteryl ends, can anchor to the outer hydrophilic part of the bilayer, while chains form a “mushroom” or “brush” conformation that changes the rigidity of the bilayer.^21^ Extending to longer blocks such as PEO–PPO (poly(propylene oxide)) diblock copolymers or PEO–PPO–PEO triblock copolymers, also known as poloxamers, introduce the opportunity to manipulate the liposomes from inside the bilayer and from the aqueous phase.^8,9,22^ Poly(oxyethylene alkyl ethers) (C_*i*_E_*j*_) or *n*-alkyl PEO polymers are at an intermediate position between PEO and diblock copolymers such as PEO–PPO. These *n*-alkyl PEO polymers consisting of hydrophilic poly(ethylene oxide) chains connected to simple hydrophobic alkyl chains are nonionic surfactant-like polymers that can show multiple self-assembled structures.^23−25^ These are commonly used in soaps and detergents in combination with other surfactants. Recently, they have also picked up momentum in biomedical applications.^26,27^ Polymers with *n*-alkyl PEO-type structures are extensively used in cosmetics, personal care, and consumer products as emulsifiers and surfactants. The nonionic nature combined with the amphiphilic nature allows these polymers to exhibit unique properties depending on environmental conditions such as the hydrophilic–lipophilic balance (HLB), pH, temperature, ionic strength, and lipid headgroups in interactions.^28^

In previous studies, it has been shown that nonionic surfactants can influence lipid bilayers in numerous ways.^29−31^ For instance, detergents such as Triton X-100 have been shown to modify the ion permeability of lipid membranes.^32^ Similarly, *n*-alkyl PEO polymers which belong to the class of nonionic surfactants can cause several modifications of phospholipid bilayers. The work of Liu *et al*. has shown that *n*-alkyl PEO polymers can insert into phospholipid bilayers by their hydrophobic end using quartz crystal microbalance (QCM) experiments.^10^ The chain length of the hydrophobic segment is known to play a key role in determining the successful insertion into the bilayer.^8,33^ In the work by Gutberlet *et al*. using POPC and C_12_E*_n_* (*n*⩽ 6, 8) systems, they have shown that the molar ratio of polymeric surfactant can decrease the thickness of the bilayer and gradually transform the system to different micellar structures.^11^

This study investigates the structural and dynamic changes of the lipid membrane caused by blending aqueous solutions of poly(ethylene oxide)-mono-*n*-octadecyl ether, C_18_-PEO4, and DOPC (1,2-dioleoyl-*sn*-glycero-3-phosphatidylcholine). This implies that C_18_-PEO4 is added to the solution of liposomes. C_18_-PEO4 consists of an *n*-octadecyl hydrophobic alkyl tail and a ∼4 kg/mol hydrophilic PEO chain (PEO4 ∼4000 g/mol). Similar polymers have been shown to play key roles in interactions with membranes for integral proteins^34,35^ and to slow lipid and vitamin E oxidation.^36^ These polymers are known to form stable micellar structures in aqueous solutions.^37,38^ We determined the influence of C_18_-PEO4 polymers on the nanoscale structure and dynamics of phospholipid membranes, which is useful for a fundamental understanding of vesicle self-assembly and enhancing drugs or nutraceutical delivery.

## Theoretical Background

In this section, we present the data modeling theory used to understand the macroscopic scattering cross section, dΣ/dΩ, for the vesicle structure and the membrane. Both the SANS and SAXS experiments are performed at an ambient temperature of 20 °C, which corresponds to the fluid phase of DOPC.^39^

### Vesicle Structure

A modified core–shell model is used to describe the vesicle form factor.^40−42^ As illustrated in Figure S1, the core is filled with water which is encapsulated by *N* shells of lipids and *N* – 1 layers of solvent in the case of multilayer vesicles (MLVs). The thickness and scattering-length density of each shell are assumed to be identical. The corresponding form factor is given by
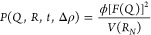
1with
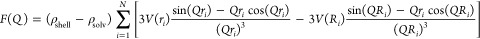
2For
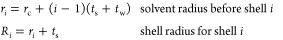
3Here, *V*(*r*) is the volume of the sphere with radius *r*, *r*_c_ is the radius of the core, *t*_s_ is the thickness of the individual shells, *t*_w_ is the thickness of the interbilayer water, and ϕ is the corresponding lipid volume fraction. The outer perimeter radius is given by *R*_SANS_ = *r*_c_ + *Nt*_s_ + (*N* – 1)*t*_w_. For DOPC, we used the neutron scattering-length density (NSLD) of the shell, *ρ*_shell_ = 3.01 × 10^9^ cm^–2^, and for D_2_O, we used the scattering-length density of the solvent, *ρ*_solv_ = 6.36 × 10^10^ cm^–2^.^43^ The macroscopic scattering cross section is obtained from the scattering intensity in SANS and is given by
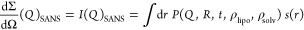
4For the size polydispersity, *s*(*r*), we used a Schulz distribution and a log-normal distribution. In addition, the thicknesses of the shell and the solvent are convoluted with a Gaussian distribution function to account for the thickness polydispersity.

### Membrane Structure

The random lamellar sheet consisting of the heads and tails of the phospholipids can be modeled using the Caille structure factor.^44−46^ SAXS provides direct access to the macroscopic scattering cross section given by the scattering intensity, and for a random distribution of the lamellar phase, it is given by
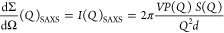
5with the scattering volume, *V*, and the distance to the lamellae, *d*. The form factor is given by
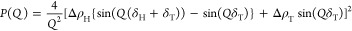
6The scattering contrasts for the head and tail are Δρ_H_ and Δρ_T_, respectively. The corresponding thicknesses are *δ*_H_ and *δ*_T_, respectively, as presented in Figure S1. The head-to-head bilayer thickness is given by *δ*_HH_ = 2(*δ*_H_ + *δ*_T_). The Caille structure factor is given by
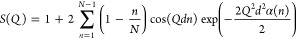
7with the number of lamellar layers, *N*, and the correlation function for the lamellae, α(*n*), defined by
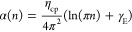
8with *γ*_E_ = 0.57721 being Euler’s constant. The elastic constant for the membranes is expressed in terms of the Caille parameter, , where κ_b_ and κ_A_ are the bending elasticity and the compression modulus of the membranes, respectively. Here, *κ*_A_ is associated with the interactions between the membranes. The position of the first-order Bragg peak is given by *Q*_0_, *k*_B_ is the Boltzmann constant, and *T* is the absolute temperature.

### Vesicle Dynamics

Neutron spin echo (NSE) spectroscopy has proven to be a powerful tool for following the molecular motions in vesicles.^47−49^ This method reaches the highest energy resolution (∼*n*eV) of all available neutron scattering spectroscopy techniques and allows us to measure the dynamic structure factor or the intermediate scattering function, *S*(*Q*, *t*), for up to several hundred nanoseconds.

Recently, it has been shown that diffusion, membrane fluctuations, the confined motion of lipid tails, and translational diffusion influence *S*(*Q*, *t*).^50^
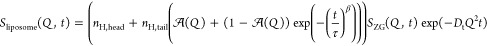
9This equation can be divided into three terms: the lipid tail motion, the membrane undulations *S*_ZG_(*Q*, *t*), and the translational diffusion, exp(−*D*_t_*Q*^2^*t*).

Variables *n*_H__,head_ and *n*_H__,tail_ relate to the relative numbers of protons in the head and the tail, respectively, and represent the contrast. In the case of *h*-DOPC, *n*_H__,head_ = 0.21 and *n*_H__,tail_ = 1 – *n*_H__,head_ = 0.79. Prefactor  refers to the elastic fraction of the lipid tail motion and is formally equivalent to the elastic incoherent structure factor (EISF) from quasielastic neutron scattering (QENS).^50^

The membrane undulations can be modeled by the Zilman–Granek (ZG) approach:^51^
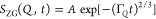
10The free parameters are the *Q*-dependent decay rate, Γ_Q_, and the amplitude, *A*.

The effective bending modulus, κ̃, is calculated from modified ZG theory by Watson and Brown^52^ as
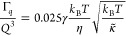
11Here, γ is a weak monotonously increasing function of κ̃/*k*_B_*T*. Unlike bicontinuous microemulsions,^53^ the effective bending modulus of the lipid membrane is ; therefore, γ = 1 is a reasonable approximation as suggested by Zilman and Granek.^51^ Here, η is the solvent viscosity, *k*_B_ is the Boltzmann constant, and *T* is the temperature on an absolute scale.

The effective bending modulus, κ̃ (or dynamic curvature modulus), is related to the bilayer curvature modulus, *κ*_*η*_, given by κ̃ = *κ*_*η*_ + 2*h*^2^*k*_m_.^52^ Here, *κ*_*η*_ is the parameter of interest which can be obtained from NSE. The monolayer area compressibility modulus for uniform plates of monolayers can be related to the monolayer bending rigidity, *κ*_b_, as *k*_m_ = 12*κ*_b_/*h*^*t*^2^^.^54^ Here, *h*_t_ is the thickness of the tail-only region of the membrane (monolayer hydrocarbon thickness) and *h* is the monolayer thickness or the height of the neutral surface from the bilayer midplane given by *h* = *δ*_HH_/2. To express the monolayer parameter, *κ*_m_, in terms of the bilayer parameter, *κ*_*η*_, we can use *κ*_*η*_ = 2*κ*_b_, and κ̃ can be expressed for a bilayer as κ̃ = *κ*_*η*_{1 + 48(*h*/2*h*_*t*_)^2^ }.^55^ Considering the neutral surface as the interface between the hydrophilic headgroup and the hydrophobic tail (*h = h*_t_),^56−60^ we can redefine eq 11 to obtain the bending rigidity of a bilayer from ULV^55^
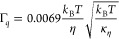
12Equation 12 has been successfully used to calculate *κ*_*η*_ for ULVs from NSE.^41,46,55,61^

For the special case of four monolayers (i.e., two bilayers or *N* = 2 (neglecting any elastic effects from interbilayer water since they are predominantly viscous)), we can redefine *κ*_*η*_ = 4*κ*_b_ and κ̃ = *κ*_*η*_{1 + 24(*h*/2*h*_t_)^2^ }, which results in
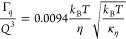
13In general, for *N* layers (i.e., 2*N* monolayers), we have *κ*_*η*_ = 2*Nκ*_b_ and κ̃ = *κ*_*η*_(1 + 12/*N*), which results in
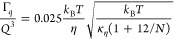
14According to this expression, the effective bending modulus for MLVs increases by a factor of  for *N* ≥ 2.

Additionally, we can analyze the mean-squared displacement (MSD) ⟨Δ*r*(*t*)^2^⟩ and the non-Gaussianity parameter  from the measured dynamic structure factor, *S*(*Q*, *t*), using a cumulant expansion given by^41,50,62,63^
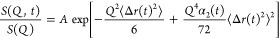
15Non-Gaussian parameter α_2_ is essentially defined as the quotient of the fourth ⟨Δ*r*(*t*)^4^⟩ and the second moment squared ⟨Δ*r*(*t*)^2^⟩^2^, and *d* = 3 is the dimension of space.^41,63,64^ Following eqs 10, 12, and 15, we can express the membrane rigidity for ULVs as a function of Fourier time, given by^50^
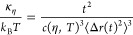
16with . The ZG approximation ⟨Δ*r*(*t*)^2^ ⟩ ∝ *t*^2/3^ and the bending rigidity as a function of time should yield *κ*_*η*_/*k*_B_*T* ∝ *t*^2^/*t*^2^ = constant. Any deviation from this constant behavior will reflect additional processes that are not considered in the ZG model. For MLVs, prefactor *c*(η, *T*) needs to be modified following eq 14.

## Experimental Section

### Sample Preparation

All chemicals and reagents were used as received. 1,2-Di(octadecenoyl)-*sn*-glycero-3-phosphocholine (DOPC) was purchased from Avanti Polar Lipids (Alabaster, AL, USA), and D_2_O was received from Sigma-Aldrich (St. Louis, MO, USA). We have used fully deuterated poly(ethylene oxide)-mono-*n*-octadecyl ether, *d*-C_18_-*d*-PEO4, to reduce scattering contributions resulting from the contrast between solvent and polymer. The polymer was synthesized by living ring-opening, anionic polymerization of fully deuterated ethylene oxide, *d*-EO. The initiator was a mixture of deuterated 1-octacosanol and corresponding potassium 1-octacosanolate. Exact polymerization conditions can be found in ref (37). The polymer was characterized by size-exclusion chromatography using a combination of the refractive index and an 18-angle light scattering detector (Optilab rEX and DAWN HELEOS-II, Wyatt) for absolute molecular weight characterization. For separation, three Agilent Plus Pore GPC columns with a continuous pore size distribution were used with a mixture of tetrahydrofuran, dimethylacetamide, and acetic acid as the eluent at a flux rate of 1 mL/min. The degree of polymerization of *d*-PEO is 92, and the dispersity index is 1.03. The PEO block has a number-average molecular weight of *M*_n_ = 4420 g/mol.

DOPC vesicles were prepared by dissolving DOPC lipid powder in chloroform and removing the solvent using a rotary evaporator and drying further under vacuum overnight. The dried lipid was hydrated using D_2_O, and the resultant solution was subjected to freeze–thaw cycling by alternatingly immersing the flask in the water at around 50 °C and placing it in a freezer at −20 °C in 10 min intervals. Finally, the solution was extruded using a miniextruder (Avanti Polar Lipids, Alabaster, AL, USA) through a polycarbonate membrane with a pore diameter of 100 nm (33 passes) to obtain unilamellar vesicles (Figure S2). Vesicle solutions were mixed with *d*-C_18_-*d*-PEO4 solutions to obtain the desired external polymeric concentrations. This technique ensures the addition of the polymer from outside the vesicles. Measurements for each mixture were averaged starting 24 h after sample preparation. All experiments were conducted at ambient temperature.

### DLS

Dynamic light scattering (DLS) measurements were performed using a Malvern Zetasizer Nano ZS (He–Ne laser wavelength, λ = 633 nm at 30 mW laser power, at a backscattering setup angle of θ = 173°. The hydrodynamic radius, *R*_h_, of the liposomes in each *d*-C_18_-*d*-PEO4 concentration was calculated from the translational diffusion coefficient, *D*_t_, using the Stokes–Einstein equation, *R*_h_ = *k*_B_*T*/(6*πη*_0_*D*_t_), with the Boltzmann constant, *k*_B_, the temperature, *T*, and the viscosity of the solvent, η_0_. DLS measurements were performed in triplicate for each mixture and averaged. In Figure S3 and Table S1, the results from DLS are reported for 5 wt % DOPC along with three different concentrations of *d*-C_18_-*d*-PEO4 polymer added to the 5 wt % DOPC liposome solution. To calculate the corresponding *R*_h_, we have used the viscosity of 5 wt% DOPC as the solvent viscosity, η_0_ = (1.6 ± 0.03) × 10^–3^ Pa·s (cf. Supporting Information).

### Cryo-TEM

Cryogenic-transmission electron microscopy (cryo-TEM) images were recorded on a Tecnai G2 F30 operated at 150 kV. A volume of 10 μL of the DOPC vesicles or DOPC polymer mixture sample was applied to a 200-mesh lacey carbon grid mounted on the plunging station of an FEI Vitrobot, and excess liquid was blotted for 2 s by the filter paper attached to the arms of the Vitrobot. The carbon grids with the attached thin films with aqueous solutions of vesicles were plunged into liquid ethane and transferred to a single tilt cryo-specimen holder for imaging. By quick plunging into liquid ethane, the vesicles are preserved in their hydrated state present at room temperature. Cryo-TEM images were obtained in the bright field setting. The DOPC vesicle concentration was maintained at 0.25 wt % or below to suit sample preparation and facilitate visualization. For vesicle–polymer mixtures, cryo-TEM images were taken for 0.25 wt % DOPC + 1 wt % C_18_-PEO4, and another set of images were taken with 0.125 wt % DOPC and 0.03 wt % C_18_-PEO4 by maintaining the DOPC/polymer weight ratio of ∼5:1 comparable to the ratio used for other techniques. All size analyses on cryo-TEM images were carried out using ImageJ software.

### SANS

Small-angle neutron scattering (SANS) experiments were conducted at the NG 7 SANS instrument of the NIST Center for Neutron Research (NCNR) at the National Institute of Standards and Technology (NIST).^65^ The sample-to-detector distances, *d*, were set to 1, 4, and 13 m at a neutron wavelength of λ = 6 Å. Another configuration with lenses at *d* = 15.3 m and λ = 8 Å was used to access low *Q* values.^66^ This combination covers a *Q* range from 0.001 to 0.6 Å^–1^, where *Q* = 4π sin(θ/2)/λ with a scattering angle of θ. A wavelength resolution of Δλ/λ = 14% was used. All data reduction to intensity, *I*(*Q*), vs momentum transfer, *Q* = |Q⃗|, was carried out following the standard procedures that are implemented in the NCNR macros to the Igor software package.^67^ The intensity values were scaled to absolute units (cm^–1^) using a direct beam. D_2_O as the solvent and empty cells were measured separately. The polymer solution composed of 1 wt % *d*-C_18_-*d*-PEO4 polymer in D_2_O was measured separately and subtracted as the background.

### SAXS

Small-angle X-ray scattering (SAXS) experiments were conducted at the LIX beamline at National Synchrotron Light Source II, Brookhaven National Laboratory, and at the Bio-SAXS beamline at the Stanford Linear Accelerator Center (SLAC) facility. The samples were measured in a flow cell to avoid damage due to the intense photon beam, with an acquisition time of 1 s. The recorded intensities were corrected for dark current, empty cell, and solvent (buffer) using standard procedures.^68,69^ The polymer solution composed of 1 wt % *d*-C_18_-*d*-PEO4 polymer in D_2_O was measured separately and subtracted as the background.

### NSE

Neutron spin echo (NSE) spectroscopy has been used to examine the effects of membrane dynamics simultaneously over broad length and time scales. We obtained NSE data at BL-15 at the Spallation Neutron Source of the Oak Ridge National Laboratory, Oak Ridge, TN.^70^ We used Hellma quartz cells with a 2 mm sample thickness. The lipid concentration was always 5%. Measurements were conducted using a wavelength of 8 Å. The BL15 ECHODET software package was used for data reduction. D_2_O and solutions of 1 wt % *d*-C_18_-*d*-PEO4 and D_2_O were measured separately and used for background subtraction.

## Results and Discussion

We first evaluated the size, shape, and morphology of the vesicle from DLS, cryo-TEM, and SANS, whereas the lipid bilayer structure is determined from SAXS. This helps us to examine the number of bilayers in the vesicle, the amount of polymer interacting with the lipid bilayer, and the effect of polymer deposition on the outer layer of the vesicle. SAXS and cryo-TEM results are used to quantify the effect of polymer-induced perturbation of the liposome structure. Next, we measure the dynamics of the phospholipid membrane by NSE to determine the effect of change in the structure on the membrane rigidity. All of these results are brought together to comprehend the mechanism of polymer-induced transformation of the vesicle structure and dynamics.

### Structure and Morphology

The vesicle size is determined by analyzing the intensity autocorrelation function, *g*^2^(*Q*, *t*), measured by DLS using a single-exponential decay:
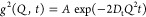
17The corresponding diffusion coefficients and hydrodynamic radii are summarized in Table S1 for different concentrations of *d*-C_18_-*d*-PEO4 polymers in 5 wt% DOPC. The corresponding autocorrelation functions and diffusion distributions are also reported in the Supporting Information (SI). While the 1 wt % *d*-C_18_-PEO4 samples have a larger hydrodynamic radius (68 ± 2 nm), lower concentrations of 0, 0.25, and 0.5 wt % have little to no influence on the size (60, 62, and 61 nm with ±2 nm error). The width of the log-normal distribution is constant within the experimental accuracy.

**Figure 1:**
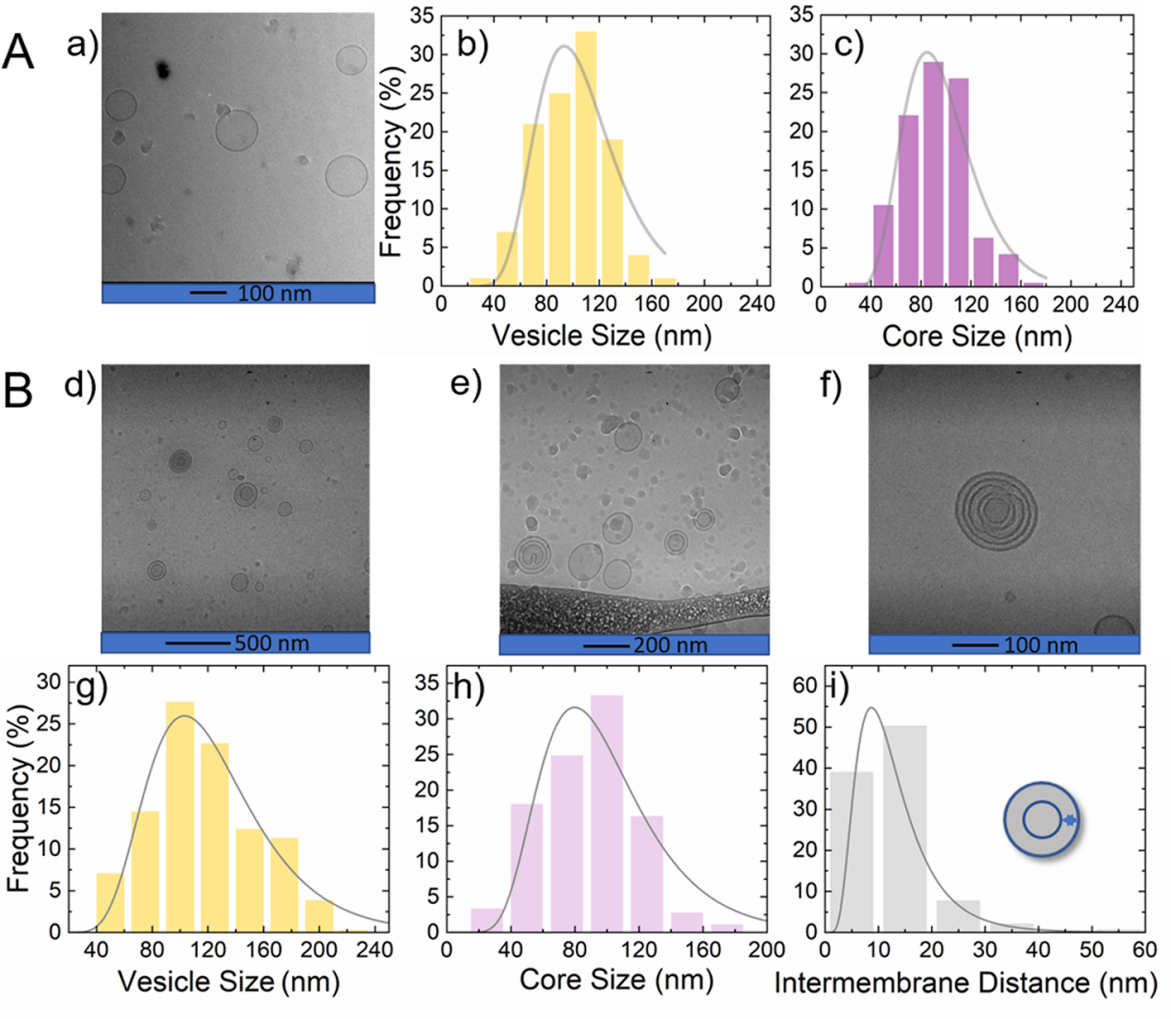
(A) Cryo-TEM analysis of 0.25 wt % pure DOPC vesicles. (a) Cryo-TEM image for pure DOPC vesicles, (b) log-normal distribution of the corresponding size distribution, and (c) the inner water core size. (B) Cryo-TEM analysis for 0.125 wt % DOPC vesicles with 0.03 wt % *d*-C_18_-*d*-PEO4 polymer (lipid/polymer w/w ratio 5:1). (d–f) Cryo-TEM images of the vesicle–polymer mixture, (g) corresponding log-normal size distribution of the vesicle size, (h) the innermost water core size, and (i) the interbilayer water thickness.

**Figure 2:**
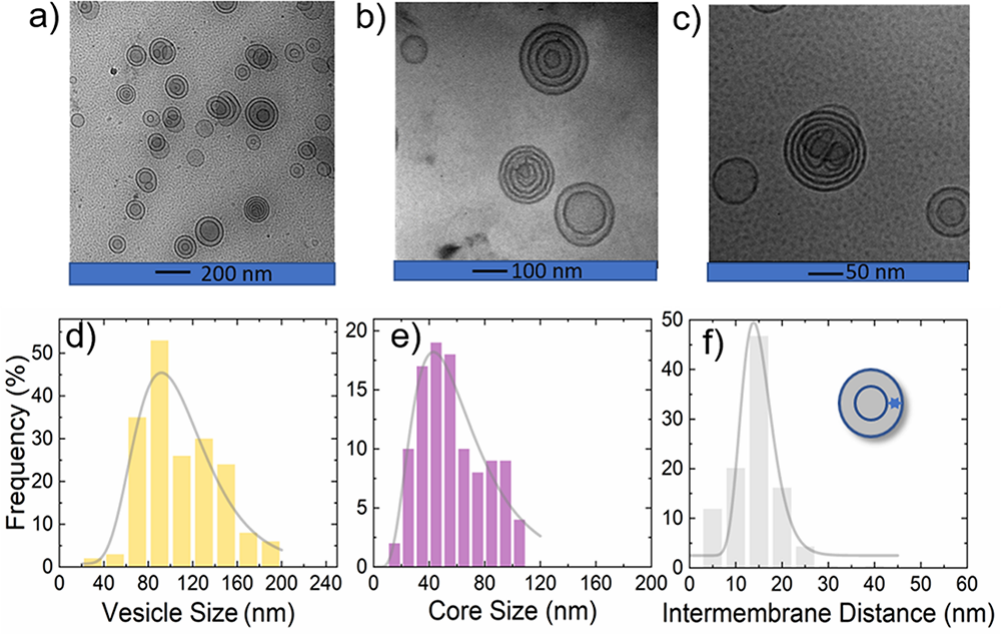
Cryo-TEM images and analyses for 0.25 wt % DOPC with 1 wt % *d*-C_18_-*d*-PEO4 in D_2_O. (a–c) Cryo-TEM images at different magnifications and (d–f) size analyses. The solid lines represent log-normal size distributions of (d) vesicle size, (e) core size, and (f) intermembrane distances, respectively. The data are tabulated in Table 1.

The morphology of the vesicles was observed using cryo-TEM. Figures 1 and 2 summarize cryo-TEM images and analyses for DOPC vesicles and DOPC vesicles in the presence of polymer. Figure 1(A-a) illustrates the cryo-TEM for 0.25 wt % pure DOPC vesicles in D_2_O. Figure 1(A-b,c) shows the respective vesicle size and water core size analysis obtained using log-normal fits. The images and size analysis of pure DOPC vesicles presented in Figure 1(A-a–c) are based on our earlier data.^41^ Because cryo-TEM experiments require low concentrations for sample preparation, 5 wt % DOPC vesicles in the presence of 1 wt % *d*-C_18_-*d*-PEO4 cannot be observed for direct comparison with SAXS and SANS. However, we measured a 0.125 wt % DOPC and 0.03 wt % *d*-C_18_-*d*-PEO4 mixture maintaining the ratio between lipids and polymers similar to that in SAXS and SANS experiments, ∼5:1 (w/w). Figure 1(B-d–f) shows cryo-TEM images of 0.125 wt % DOPC vesicles dispersed in 0.03 wt % *d*-C_18_-*d*-PEO4 polymer solution. These indicate the presence of a highly polydisperse mixture of unilamellar and multilamellar vesicles (MLV). The data analysis yields a log-normal size distribution for the vesicle size, innermost water core size, and interbilayer water thickness as shown in Figure 1 B-g–i, respectively.

Because of the low count of vesicles in the cryo-TEM sample maintained at a 5:1 lipid weight ratio, we further explored 0.25 wt % DOPC with 1 wt % *d*-C_18_-*d*-PEO4 polymer data summarized in Figure 2 where the *d*-C_18_-*d*-PEO4 concentration is similar to that determined from the other techniques described. In both cases, multilamellar vesicle formation and increasing vesicle size were observed.

The corresponding vesicle perimeter radius yields *R*_p__,TEM_ = 52 ± 4 nm with 34 ± 9% polydispersity. The water core size analysis yields a core radius of *R*_c__,TEM_ = 28 ± 1 nm with 52 ± 5% polydispersity. Figure 2(f) shows the average interbilayer water thickness of *t*_w__,TEM_ = 14.6 ± 0.5 nm and its polydispersity of 23 ± 3%. While the addition of the polymer leaves the perimeter diameter slightly increased compared to the pure DOPC vesicles with *R*_p__,TEM_ = 51 ± 3 nm and 30 ± 7% polydispersity, the core size is substantially changed compared to the pure DOPC vesicles with *R*_c__,TEM_ = 47 ± 1 nm and 30 ± 6% polydispersity. This significant reduction in the size of the water core in the presence of *d*-C_18_-*d*-PEO4 comes with a substantial increase in polydispersity. For a more direct comparison, Table 1 summarizes the values. We obtained *R*_p__,TEM_, *R*_c__,TEM_, and *t*_w__,TEM_ values by counting as many as 187, 106, and about 278 distances, respectively, including different orientations. Compared to scattering experiments, this number is still low; therefore, we used scattering experiments for improved statistical representation.

**Table 1:** Structural Parameters from Cryo-TEM and SANS^a^

	cryo-TEM		SANS	
samples	0 wt % *d*-C_18_-*d*-PEO4	1 wt % *d*-C_18_-PEO4	0 wt % *d*-C_18_-PEO4	1 wt % *d*-C_18_-PEO4
*N*	1	∼3	1	2
*R*_p_ (nm)	51 ± 3	52 ± 4	54 ± 2	59 ± 2
*R*_c_ (nm)	47 ± 2	28 ± 1	51 ± 2	37 ± 2
*t*_w_ (nm)	NA^c^	15 ± 1	NA^c^	15 ± 1
*T*_s_ (nm)	NA^b^	NA^b^	3.6 ± 0.1	3.4 ± 0.2
*R*_p_ polydispersity (%)	30 ± 7	34 ± 9	30 ± 2	40 ± 2
*R*_c_ polydispersity (%)	30 ± 6	52 ± 5	30 ± 2	40 ± 2
*t*_w_ polydispersity (%)	NA^c^	23 ± 3	NA^c^	41 ± 4

a Number of layers (*N*), outer perimeter radius of the vesicle (*R*_p_), water core radius (*R*_c_), intermembrane water thickness (*t*_w_), bilayer thickness (*t*_s_), and corresponding size polydispersity.

b Not visible in TEM images.

c Not applicable to ULVs.

**Figure 3:**
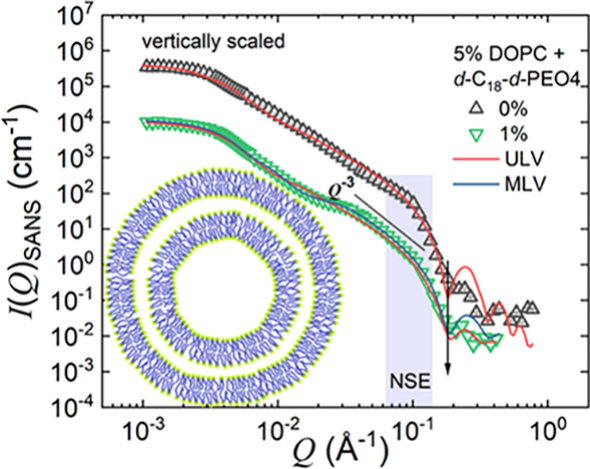
SANS scattering data for pure 5 wt % DOPC and DOPC mixed with 1 wt % *d*-C_18_-*d*-PEO4 polymer dispersed in D_2_O. The solid lines represent the ULV (*N* = 1) and MLV (*N* = 2) model described in eqs 1, 2, and 4. The shaded region depicts the *Q* range over which NSE experiments are performed. The inset depicts the schematic illustration of the MLV (*N* = 2) model. The 0 wt % data is vertically unscaled, and the DOPC mixed with 1 wt % *d*-C_18_-*d*-PEO4 data is vertically scaled, multiplied by a factor *c* (*c* = 30), for better visualization

Figure 3 presents SANS data of 5 wt % DOPC in D_2_O and 5 wt % DOPC mixed with 1 wt % *d*-C_18_-*d*-PEO4. The solid red lines represent the data modeling using the vesicle form factor as described in eqs 1, 2, and 4. For both pure vesicles and vesicles with 1 wt % *d*-C_18_-*d*-PEO4 polymer samples, we illustrate data modeling using a ULV form factor (*N* = 1). As shown in Figure 3, although we obtain a satisfactory description for the pure DOPC, we cannot explain the vesicles with 1 wt % *d*-C_18_-*d*-PEO4 data using a ULV model. We observe a *Q*^–3^ power-law dependence over the *Q* range of 0.02 to 0.08 Å^–1^, which might be due to scattering from a highly folded and convoluted surface arising from the adsorption of the polymer on the vesicle surface.^71,72^ Such surfaces are also visible in the cryo-TEM images (Figures 1B(d–f) and 2(a,b,c)) along with the formation of MLVs. In this case, the presence of the polymer facilitates MLV formation. We modeled the SANS data for the 1 wt % *d*-C_18_-*d*-PEO4-DOPC sample using the MLV model with the water core encapsulated by *N* = 2 lipid shells. The inset figure represents the SANS model used in data modeling. The parameters obtained from the TEM analysis, like the core radius, *R*_c_, and the interbilayer water thickness, *t*_w_, were used as an initial estimation for fitting to obtain the outer perimeter radius *R*_p_ as presented in Table 1. The data fitting was done with a maximum of two or three free parameters at a time. We varied each of the parameters systematically again and again in a loop until a negligible change in the fitted curve and residual had been achieved. We covered all of the parameters reported in Table 1, where the error bars represent the maximum deviation for each parameter over which our model can describe the data. Therefore, they represent the uncertainties in the parameters. The plots of residuals for the fits are included in the SI.

As suggested by DLS and cryo-TEM experiments, we used a log-normal distribution for the polydispersity. From SANS, we obtain a 9% increase in the size of the DOPC vesicles, *R*_SANS_ = 53.6 ± 0.2 nm (0 wt % *d*-C_18_-*d*-PEO4) to 57.9 ± 0.5 nm (1 wt % *d*-C_18_-*d*-PEO4). In the presence of 1 wt % *d*-C_18_-*d*-PEO4 polymer, along with the formation of MLVs we observe an ∼5% reduction in the bilayer thickness and find an interbilayer water layer of *t*_w_ ≈ 14 nm. Both SANS and cryo-TEM yield a large polydispersity of *t*_w_.

An estimate of the amount of polymer interacting with phospholipid vesicles was obtained by additional SANS experiments. Figure S9 in the SI illustrates SANS data for (i) 0.25 wt % DOPC in D_2_O, (ii) 5 wt % of *h*-C_18_-*h*-PEO4 in D_2_O, and (iii) 0.25 wt % DOPC with 5 wt % *h*-C_18_-*h*-PEO4 in D_2_O (mixture). The black line represents the weighted sum of intensities of samples (i) and (ii). While these results indicate that the blend of *h*-C_18_-*h*-PEO4 and DOPC has characteristic features of the micellar structure factor and the unperturbed DOPC liposomes, deviations indicate interactions between polymer and liposome. The data in Figure S9 is particularly useful because the weighted addition tells us that the 5 wt % polymer fraction that forms micelles is reduced to 4.7 wt %. Since the critical micellar concentration (CMC) of *h*-C_18_-*h*-PEO4 is 0.01 wt %,^73^ we calculated that 0.3 wt % of the polymer interacts with 0.25 wt % of the DOPC vesicles. This number accounts for 6% of the added *h*-C_18_-*h*-PEO4. It is very likely that the system of micelles and liposomes dynamically exchanges unimers. In this way, 94% of the unimers form micelles, and 6% refers to the number of molecules that are dynamically exchanging. Hence, this 6% refers to the maximum amount, while the average amount of polymer inserted in the liposome can be significantly lower. This information is used to calculate the lipid/polymer ratio in the lipid layer and contrast with conditions arising later.

**Figure 4:**
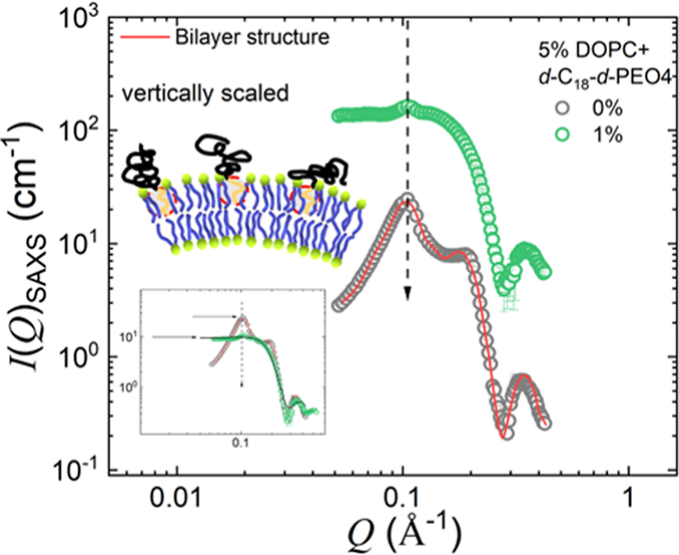
SAXS diffraction data for pure DOPC and DOPC mixed with a 1 wt % *d*-C_18_-*d*-PEO4 polymer dispersed in D_2_O. The solid red line is the data modeled using the MLV form factor. The data is vertically scaled. The inset depicts the unscaled data. The schematic illustration of the bilayer structure with the polymers wedged in is depicted. The 1 wt % data is vertically scaled by a factor of *c* = 15 for proper visualization. Further SAXS data analysis is in the SI.

Figure 4 compares SAXS on 5 wt % DOPC vesicles in D_2_O with 5 wt % DOPC and the 1 wt % *d*-C_18_-*d*-PEO4 mixture. Represented by the solid red line, the aqueous solution with only DOPC is modeled by the Caille structure factor of multilamellar vesicles (eqs 5, 6, and 7). We find *N* = 2 layers with a lamellar repeat distance *d* = 6.6 ± 0.4 nm and a head-to-head bilayer thickness δ_HH_ = 4.5 nm. We point to the observation of *N* = 2 (SAXS) and 1 (SANS) layers, though we use the same sample in SAXS and SANS. There is no indication that the sample has been affected by any radiation damage as we used a flow cell. However, the experiments of Courbin *et al*.^74^ suggest the formation of multilamellar vesicles by shear. This is certainly not any proof of our observation, but it could point to more detailed investigations of flow-induced effects. A recent paper by Heberle *et al*. discusses another possibility where a small percentage of MLVs may be present even without being subjected to flow. They have shown that even after extrusion through polycarbonate membranes to synthesize unilamellar vesicles, a small fraction of MLVs may be present, and this will give rise to a SAXS profile that is intermediate between MLVs and ULVs even with a small percentage of MLVs.^75^ Therefore, this could be another reason for the observation of *N* = 2 (SAXS) and 1 (SANS) layers for pure DOPC vesicles.

In the presence of *d*-C_18_-*d*-PEO4 polymers, the most obvious observation is the substantially decreasing intensity of the peak at *Q* ≈ 0.1 Å^–1^ (vertical dashed arrow). The inset shows that the second peak (or shoulder) roughly at *Q* ≈ 0.2 Å^–1^ disappears as well. A separation of the intensity in the product of the structure and form factor (Figure S10 in the SI) suggests that the observed changes arise from a perturbation of the lamellar repeat distance, *d*, which indicates a more heterogeneous system, supporting the independent information by cryo-TEM.

We used SANS results summarized in Table 1 to calculate the number of lipids and the average cross-sectional or lateral space that lipid molecules assume in distinct leaflets. The total number of lipids (per liposome) *N* is given by, *N* = ∑_*i*_*N*_*i*_, with *N*_*i*_ being the number of lipids in each leaflet *i*. Our system is bilamellar, thus amounting to four leaflets and hence *N* = *N*_1_ + *N*_2_ + *N*_3_ + *N*_4_.

If we assume that the cross-sectional area of one lipid, α, is known, then , with *A*_*i*_ referring to the surface area of one leaflet. The liposome surface area is *A*_*i*_ = *πR*_*i*_^2^, with *R*_*i*_ being the radius, which is known from SANS. To estimate the number of lipids in each leaflet, we assume that the area per headgroup, α, is independent of the curvature of the leaflet. Because the radii are only slightly different, this approximation works very well, as indicated by the results. In the main text, we report the core radius, *R*_c_, the thickness of the bilayer, *δ*_HH_, and the thickness of the water layer, *t*_w_ (cf. Table 1). In this way, we identify *R*_1_ = *R*_c_, *R*_2_ = *R*_c_ + *δ*_HH_, *R*_3_ = *R*_c_ + *δ*_HH_ + *t*_w_, and *R*_4_ = *R*_c_ + 2*δ*_HH_ + *t*_w_. Using , we arrive at *N*_1_*a* = 4*πR*_c_^2^, *N*_2_*a* = 4π(*R*_c_ + *δ*_HH_)^2^, *N*_3_*a* = 4π(*R*_c_ + *δ*_HH_ + *t*_w_)^2^, and *N*_4_*a* = 4π(*R*_c_ + 2*δ*_HH_ + *t*_w_)^2^.

As
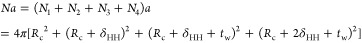
we obtain  and *N*_1_ = 23 000 ± 1200, *N*_2_ = 27 900 ± 1500, *N*_3_ = 52 500 ± 3900, and *N*_4_ = 59 000 ± 4400 with *N* = 163 100 ± 11 200 as estimates of the number of lipids in each leaflet and one vesicle.

If we assume a lipid headgroup to be circular, then *a* = *πr*^2^ yields the radius *r* = 0.48 ± 0.07 nm or diameter *d* = 0.96 ± 0.15 nm a lipid occupies. It should be noted that a lipid head surface area of 0.69 ± 0.02 nm^2^ was obtained in our previous work,^41^ yielding *r* = 0.47 ± 0.01 nm. From the SAXS study, an area of 0.72 ± 0.005 nm^2^ was reported,^76^ yielding *r* = 0.47 ± 0.01 nm. These results agree very well. Furthermore, in neutron spin echo or quasi-elastic neutron scattering experiments, the lipid tail motion has been analyzed by assuming that the lipid tails relax in an environment that is described by a cylindrical potential with a cylinder radius of approximately 0.43 nm.^50^ This number is very similar to the radius obtained by the entirely independent estimate via the number of lipids per leaflet. Therefore, all experiments seem to be in favor of lipids that occupy on average a cylindrical area with the diameter being roughly 1 nm.

Using the above information and SANS data on DOPC and *h*-C_18_-*h*-PEO4, the relative fraction of polymer in the mixture of 5 wt % DOPC vesicles with 1 wt % *d*-C_18_-*d*-PEO4 is attempted. As explained in the SI (*N* = 2, page S7), considering that all polymers interact with only the outermost leaflet, out of a total of 5 wt % DOPC lipids in 1 mL = 6.36 × 10^–5^ mol × 6.02 × 10^23^ mol^–1^ = 3.83 × 10^19^ lipids, the number of lipids in the total outer leaflets per milliliter was 1.39 × 10^19^. Since there are 2.34 × 10^14^ vesicles per mL, we obtain 1.24 × 10^18^*d*-C_18_-*d*-PEO4 per mL inserted into the outer layer.

As shown on SI page S7, Figure S9, if we compare fully hydrogenated samples, the molar ratio for 0.25 wt % DOPC to 0.3 wt % *h*-C_18_-*h*-PEO4 is 2:1, which interacts with the outer leaflet of the outer lipid bilayer. We argued that there might be a dynamic exchange, hence 0.3 wt % interacts with the bilayer, while 4.7 wt % of the polymers form micelles and stay in the aqueous phase. The molar ratio in the case of 5 wt % DOPC to 1 wt % *h*-C_18_-*h*-PEO4 is 11:1. This suggests that all of the 1 wt % polymers interact with the outer leaflet of the vesicle because the saturation level of 5.6 wt % is not reached when excess polymers are present to form micelles. Thus, we have a lipid fraction of 0.91 or a polymer fraction of 0.08 if we consider only the outer leaflet, and we would assume that all polymers are in this outer leaflet. If the polymer interacts equally with all four leaflets, then this number would be 4 times lower and would be less than 0.02. Whenever we assume that all of the polymer inserts in the vesicles, the concentration range for the outer leaflet ranges from 0.02 to 0.08. The polymer concentration is very low compared to the lipid concentration. Therefore, it is in favor of approximating the cross-sectional area, *a*, of a lipid by a constant value.

### Dynamics

**Figure 5:**
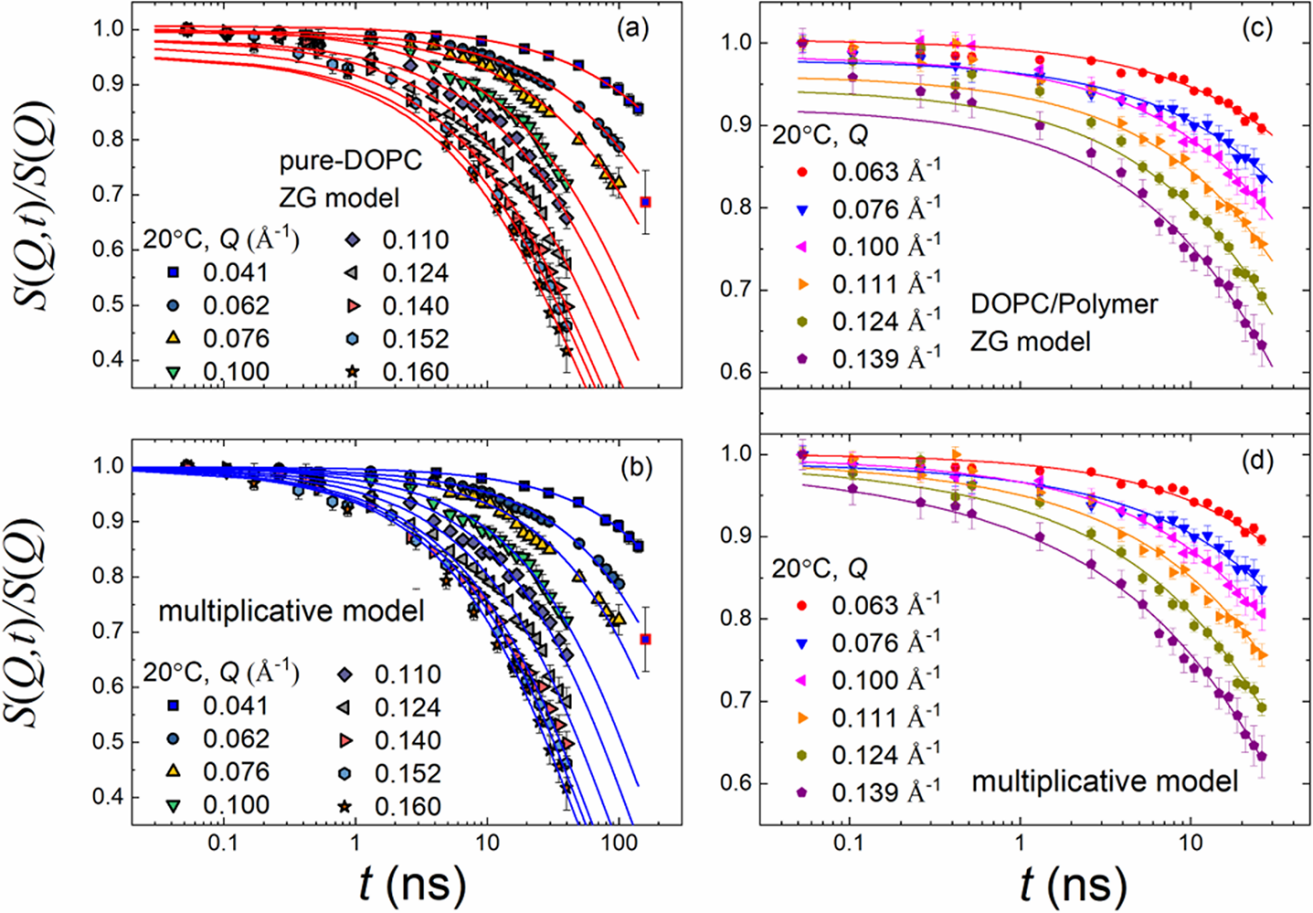
Lin–log representations of the normalized dynamic structure factor, *S*(*Q*, *t*)/*S*(*Q*), as a function of Fourier time, *t*, for different *Q*’s, pure DOPC vesicles (a, b), and DOPC mixed with 1% mass fraction of *d*-C_18_-*d*-PEO4 polymer dispersed in D_2_O at 20 °C (c, d). The same data sets are analyzed by fits using the Zilman Granek model (eq 10) and the multiplicative model described in eq 9 that includes diffusion and confined motion. The error bars represent one standard deviation. A lin–lin plot for DOPC mixed with polymer is presented in the SI. The data for pure DOPC were reproduced from our previous studies.^41,50^

Figure 5(c) illustrates the dynamic structure factor, *S*(*Q*, *t*), measured by NSE for the blend of the aqueous (D_2_O) solutions with 1 wt % *d*-C_18_-*d*-PEO4 and 5 wt % DOPC, covering a *Q* range from 0.063 to 0.139 Å^–1^. The solid lines represent the description by the ZG model (eq 10). The logarithmic time axis accentuates deviations at low Fourier times and indicates processes beyond membrane undulations. The membrane rigidities, *κ*_*η*_/(*k*_B_*T*), as calculated from eq 13 (for *N* = 2 layers) are listed in Table 2. For a better comparison, we have included the results of pure DOPC (*N* = 1) from our previous studies in Figure 5(a,b).^41,50^

**Table 2:** Membrane Rigidity *κ*_*η*_ Obtained for a 1 wt % *d*-C_18_-*d*-PEO4-DOPC Sample Using Different Models for *D*_t_ = 0 and η = *η*_D___2_O_^a^

κ_η_/*k*_B_*T*						
parameters	concentration *d*-C_18_-PEO4, wt %	*N*	ZG analysis (full-time range) (eq 10)	ZG analysis (*t* > 5 ns)	multiplicative model (eq 9)	MSD analysis
*D*_t_ = 0	0	1	26 ± 1	20 ± 2	21 ± 2	18 ± 2
η = η_D_2_O_	1	2	26 ± 5	28 ± 5	29 ± 5	30 ± 3
	1	1	13 ± 3	14 ± 4	15 ± 3	15 ± 2

a We used the results of the 0 wt % *d*-C_18_-*d*-PEO4-DOPC sample from our previous study.^41,50,61^ For comparison with pure DOPC, we have included *κ*_*η*_ for *N* = 2 and 1 layers.

The observation of three different processes in neat protiated DOPC—translational diffusion of the vesicle, ZG membrane undulation, and confined tail motion—suggests using the multiplicative model (eq 9). Figure 5(d) shows better agreement. The obtained *κ*_*η*_/(*k*_B_*T*) from eq 13 is reported in Table 2.

**Figure 6:**
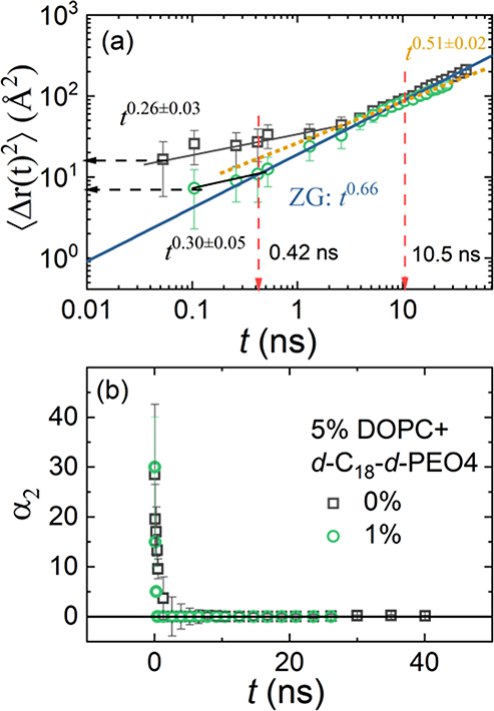
(a) Mean square displacement, ⟨Δ*r*(*t*)^2^ ⟩, vs Fourier time, *t*, for 5 wt % *h*-DOPC^41^ and 5 wt % *h*-DOPC dispersed in 1 wt % *d*-C_18_-*d*-PEO4 in D_2_O at 20*°*C. The solid lines represent the experimental power-law dependence. The horizontal dashed arrow indicates absolute values of ⟨Δ*r*(*t*)^2^ ⟩ for *t* ≤ 0.1 ns. (b) Corresponding non-Gaussian parameter α_2_.

To examine the effect of different dynamics by a model-independent approach, we have calculated the MSD, ⟨Δ*r*(*t*)^2^⟩, for the 1 wt % *d*-C_18_-*d*-PEO4-DOPC sample using eq 15 along with the non-Gaussian parameter, α_2_, as illustrated in Figure 6(a,b), respectively. For a better illustration of changes, we included earlier on pure DOPC, which shows a *t*^0.26±0.03^ power law dependence at low Fourier times, *t* < 3 ns, and finite α_2_ seems to be related to the tail motion.^41,50^ We will now discuss potential sources of these differences.

First, the ZG region (*t*^0.66^ region) stays almost constant, but the short-time region is highly affected. Such a disappearance was observed earlier in the case of contrast-matched lipid tails. Hence, we discuss the likelihood of contrast matching of the lipid tails first.

#### Contrast Matching

Three independent arguments deem contrast matching less likely. (i) A low concentration of the polymer in the bilayer. (ii) In case the tail is entirely contrast matched, there would be a visible contribution by the headgroups in Γ_q_/*Q*^3^. (iii) The contrast conditions are far from the contrast-matching conditions.

(i) Our calculations (cf. SI) suggested that for 5 wt % DOPC used in NSE the concentration range of the polymer in the outermost leaflet ranges from 0.02 to 0.08 wt %. This concentration is too low to cause any visible effect on the contrast of the NSE experiment.
(ii) There is further support that the contrast may not be matched. In the case of lipid tail contrast matched with the solvent, the scattering of the lipid headgroup prevails, which allows the observation of membrane thickness fluctuations as a peak in the normalized ZG decay rate Γ_*Q*_/*Q*^3^ vs *Q* plot.^47,61^Figure S13 in the SI illustrates *Γ*_*Q*_/*Q*^3^ = const. This suggests that the contribution of the headgroup does not prevail. The absence of the membrane fluctuations alone is not full proof that the tails are not contrast-matched. However, this experimental observation together with the absence of a peak Γ_*q*_/*Q*^3^ augments the confidence that the tail is not contrast-matched.
(iii) Finally, we ask what the contrast-matching condition would be for the lipid tail and hydrophobic block *d*-C_18_. The idea is to consider the hydrogenous lipid tails, the deuterated hydrophobic *d*-C_18_, and an aqueous solution with D_2_O and H_2_O as a quaternary system in which the contrast is determined by the difference in the average scattering length densities of the *d*/*h*-solvent and the lipid tail/*d*-C_18_. Such a simplified consideration neglects the formation of domains or rafts, which will be discussed below. For such a system, the contrast is Δρ = ρ̅_tail/*d*-C18_ – ρ̅_*d*/*h*-solvent_. The average scattering-length densities are ρ̅_tail/*d*-C18_ = ϕ_1_ρ_tail_ + (1 – ϕ_1_)ρ_*d*-C_18__ and ρ̅_*d*/*h*-solvent_ = ϕ_2_ρ_D_2_O_ + (1 – ϕ_2_)ρ_H_2_O_. The NSE experiments were conducted in D_2_O, hence ϕ_2_ = 1 and ρ̅_*d*/*h*-solvent_ = ρ_D_2_O_. The neutron scattering-length density of the tail is *ρ*_tail_ = 4.6 × 10^8^ cm^–2^, and the hydrophobic part of *d*-C_18_-*d*-PEO4 is ρ_*d*-*C*_18__ = 6.52 × 10^10^ cm^–2^.^37,43,77^ Contrast-matching conditions imply that Δρ = 0 or ρ_D_2_O_ = ϕ̃_1_ρ_tail_ + (1 – ϕ̃_1_)ρ_*d*-C_18__. Therefore, we can calculate the contrast-matching concentration from ϕ̃_1_ = (ρ_D_2_O_ – ρ_*d*-C_18__)/(ρ_tail_ – ρ_*d*-C_18__). As shown in Figure S15 of the SI, we need a mixture of 98% *d*-C_18_ chains in *h*-DOPC lipid to contrast match with D_2_O. In the samples used, the deuterated chain fraction in the bilayer is significantly lower than that.

#### Physical Meaning of the (Almost) Disappearance of the Short-Time Region

The MSD in Figure 6 illustrates the lowered contribution of the tail dynamics at low Fourier times to the MSD. It may entirely disappear, or the two values at the shortest Fourier times may indicate a small residue of the MSD of the tail motion. We use the fact that the Q-range of the NSE experiment essentially represents the length of the cylindrical confinement, as discussed in a previous publication. The summary of SANS results (Table 1) shows that the thickness of the bilayer changes only from 3.6 ± 0.1 to 3.4 ± 0.2 nm.

If we compare the mixture of DOPC with the polymer, then this is a situation similar to the case of DMPC/DOPC mixtures (i.e., mixtures of saturated and unsaturated hydrocarbons). In the case of adding DOPC to DMPC, the data seem to be in favor of stronger tail confinement.^78^ However, the low concentration of polymer inserted into the liposome observed by SANS (0.02 to 0.08) may not argue in favor of any dilution effect by adding a saturated hydrocarbon chain (polymer) to an unsaturated lipid vesicle.

The structural information together with the calculated number of polymers in the bilayer makes a change in the confinement less likely, at least in the time- and length-scale regions of the NSE experiment. Hence, we cannot reach a final conclusion, and further experiments will be necessary to understand what causes the disappearance of the short-term motion that was assigned to the tail earlier.

**Figure 7:**
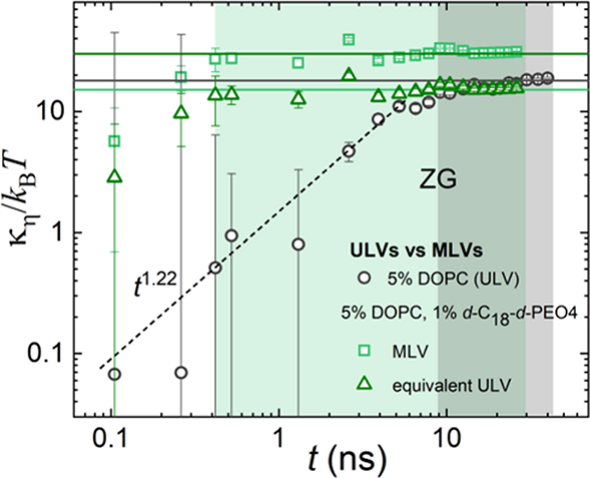
Membrane rigidity, *κ*_*η*_, divided by the thermal energy, *k*_B_*T*, with the Boltzmann constant, *k*_B_, and the temperature, *T*, as a function of Fourier time. The data is calculated over the NSE time window from the MSD in Figure 6(a) for 0 and 1 wt % *d*-C_18_-*d*-PEO4 polymer dispersed in D_2_O at 20 *°*C. The calculated average values from the flat ZG region (shaded green and gray, respectively) are illustrated by the horizontal lines. These lines represent the bending modulus, *k*/*k*_B_*T*, and the values are listed in Table 2. The different power laws are explained in the text.

We can utilize the model-free approach to obtain the changes in the membrane rigidity in the presence of the polymer. In Figure 7, the calculated *κ*_*η*_/*k*_B_*T* for a 1 wt % *d*-C_18_-*d*-PEO4-DOPC sample for MLVs with *N* = 2 is illustrated. An equivalent ULV membrane rigidity, , is used for comparison with a 0 wt % *d*-C_18_-*d*-PEO4-DOPC sample (ULV) from our previous work.^41,50^ In this approach, any deviation from the ZG model is magnified. The shaded area in Figure 7 elucidates a wider region (time-independent behavior) in the presence of 1 wt % *d*-C_18_-*d*-PEO4 polymer that behaves like the standard ZG motion.

In the limit of *Q* → 0, we have *κ*_*η*_/*k*_B_*T* ∝ *t*^2–3*x*^. In this case, the ZG prediction with *x* = 2/3 = 0.66 yields time-independent behavior (solid lines), whereas the lipid tail motion has *x* = 0.26, yielding a *t*^1.22^ contribution (dotted line). This simple estimate assumes that the effect of translational diffusion of the vesicle is negligible (*D*_t_ = 0), and α_2_ = 0. It should be noted that for a 1 wt % *d*-C_18_-*d*-PEO4 sample the additional *t*^0.51^ dependence in MSD analysis (Figure 6 (a)) for *t* > 10.5 ns has been eliminated by subtracting a *t*^0.47^ contribution following the *κ*_*η*_/*k*_B_*T* ∝ *t*^2–3*x*^ dependence, with *x* = 0.51. This yields *κ*_*η*_/*k*_B_*T* = 16 ± 2 for the 1 wt % *d*-C_18_-*d*-PEO4 sample.

In Table 2, we have compared *κ*_*η*_/*k*_B_*T* obtained from ZG (eq 10), multiplicative (eq 9), and MSD analysis (Figure 6) approaches using the D_2_O viscosity in eqs 12 and 13. We have also included the calculation from the ZG model (equation) using only the higher Fourier times (*t* > 5 ns) for the analysis, which results in *κ*_*η*_/*k*_B_*T* values similar to those obtained by the multiplicative and MSD analysis. This emphasizes that the undulations prevail in the intermediate time range. For a proper assessment of the single bilayer rigidity of the 1 wt % *d*-C_18_-*d*-PEO4-DOPC sample, we have compared with the 0% *d*-C_18_-*d*-PEO4-DOPC sample by calculating the equivalent ULV membrane rigidity, . This analysis clearly elucidates the fact that in the presence of 1 wt % *d*-C_18_-*d*-PEO4 polymer the membrane rigidity in each bilayer decreases.

Several previous studies have explored phospholipid membrane rigidity changes upon interactions with structurally or chemically similar molecules. In a 2018 study, Elsayed *et al*. have shown that the membrane rigidity can be decreased with nonionic surfactants such as the ones discussed in the Introduction as well as C_18:1_EO_20_ molecules which are structurally quite similar to the *n*-alkyl PEO polymer used in this work.^79^ Some have reported an increased bending rigidity upon interactions with block copolymers. For instance, poly(MPC-PPO-MPC) polymers have been shown to increase the phospholipid vesicle rigidity by weak dipole–dipole interactions with the zwitterions.^80^ Despite the changes in collective dynamics, there are no reported changes in the static structure of the vesicles as observed in this case. Another study by Kang *et al*. explored the use of amphiphilic triblock copolymers (PEO-PCL-PEO and PEO-PDMS-PEO) with phospholipid giant unilamellar vesicles where they observed increased membrane rigidity due to lateral coassembly.^81^ The diversity of different block copolymers therefore can impact the membrane rigidity differently, providing an excellent opportunity to manipulate this property to match different applications.

**Figure 8:**
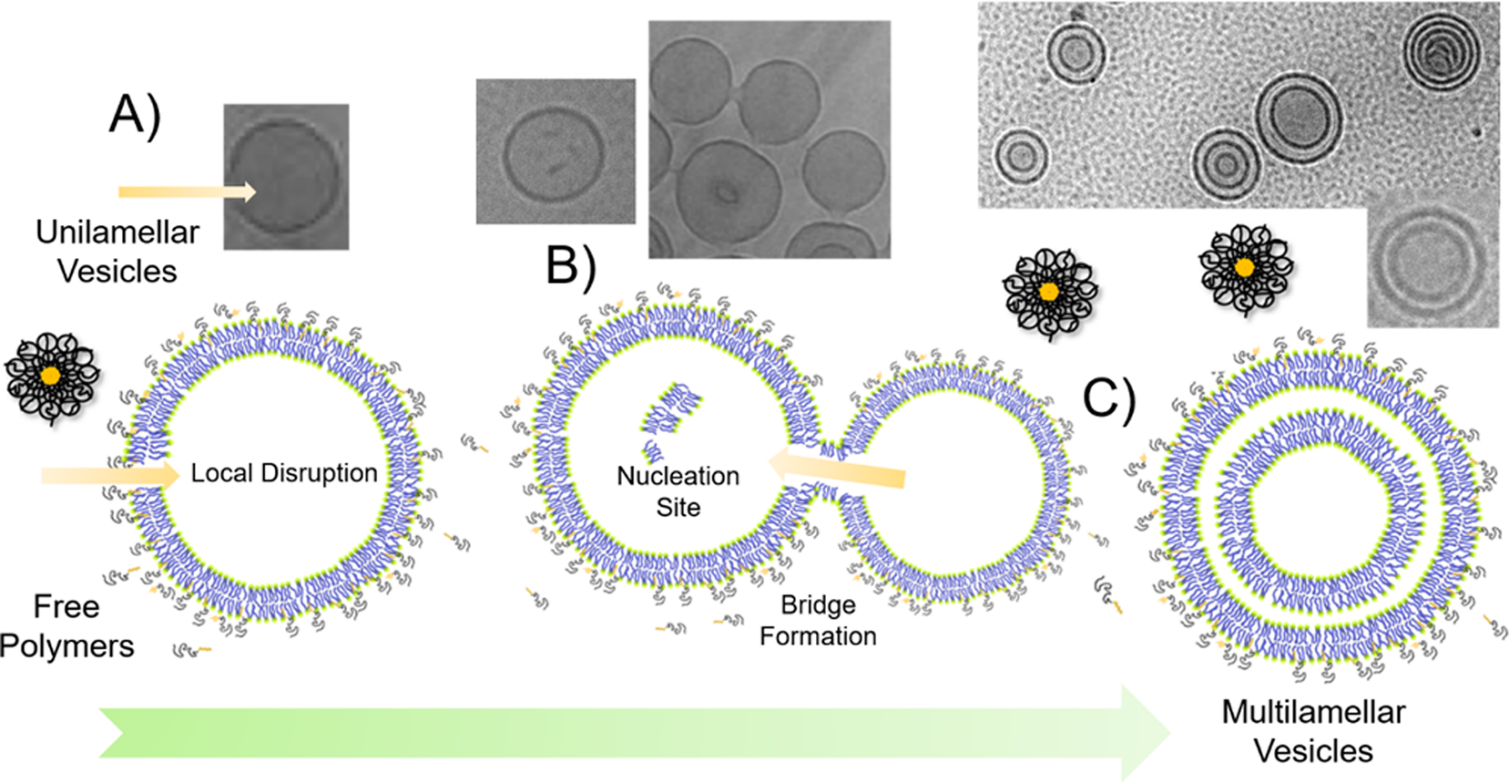
Schematic illustration of the proposed mechanism of MLV formation in (A) transient local disruption, (B) bridge formation, and (C) multilayer vesicles (MLVs) in the presence of *d*-C_18_-*d*-PEO4 polymers.

In summary, from both structure and dynamics, we have observed that a disruptive effect introduced by nonionic surfactant-like polymers causes a transformation from ULV to MLV structures. In short, the proposed mechanism of MLV formation has been illustrated in Figure 8. At first, the polymer chain prefers to slice into the bilayer membrane. This phenomenon causes structural defects in the lipid bilayers, as illustrated in Figure 8A, supported by the TEM images. This disruption provides an opportunity for bridge formation (Figure 8 B) between individual vesicles that leads to the transport of free lipids, which acts as a nucleation site for the formation of a new bilayer, resulting in the formation of MLVs (Figure 8 C) determined by SANS. A similar mechanism of the induced multilamellar structure was observed by using cell-penetrating peptides (CPPs) where the mechanism has transition steps involving similar transient pores.^82^ The presence of a free lipid layer as presented in the nucleation site has been observed in nanoemulsions.^83^ The reverse process (multi- to unilamellar transformation) has been observed in the case of lectin vesicles when subjected to diphenylalanine-based small peptides. The transition, in this case, occurs due to the formation of peptide aggregates on the membrane interface interacting with the phosphate groups. This destabilizes the fine balance of attractive repulsive forces in multilayered structures, resulting in the formation of larger unilamellar structures.^84^ Our present study is another example of a destabilization effect where a fine balance of these forces drives MLV formation.

## Conclusions

Bringing together the information from SAXS and SANS, we can illustrate the effect of mixing *d*-C_18_-*d*-PEO4 and vesicle solutions in Figure 8. The polymer forms micelles in an aqueous solution as shown by Zinn *et al*.^85^ We observed from SAXS analysis that in the presence of lipid vesicles the polymer chain consisting of hydrophobic alkyl and hydrophilic PEO segments prefers to wedge into the bilayer membrane. The hydrophobic alkyl group of the polymer resides in the lipophilic hydrocarbon core of the lipid bilayer. Such preferential migration of the unimers into the membrane was also observed for bicontinuous microemulsions.^86^ The partial disruption of the bilayer lipid order by the polymer is responsible for about a 30% reduction in membrane rigidity in each bilayer, as determined by NSE. These polymers in the bilayer have their long hydrophilic PEO chains dangling outside the vesicle interface into the water. They exert a hydrophilic tension on the lipid membrane, causing an overall expansion in the size of the vesicles, as verified by DLS and SANS. The simultaneous formation of MLVs contributes further to the increase in the size of the vesicles. These results emphasize the opportunities to use a unique hydrophilic–hydrophobic polymer acting as a hybrid of diblock and nonionic surfactants that can transform membrane structures and control their dynamics with possible applications in topical drugs or nutraceutical formulations.

## Supporting Information Available

Vesicle preparation, diffusion coefficients, cryo-TEM images, SAXS diffraction, NSE data on a linear scale, and ZG decay rate from NSE (PDF)
