# Supplementary material for: Manipulating Phospholipid Vesicles at the Nanoscale: A Transformation from Unilamellar to Multilamellar by an n-Alkyl-poly(ethylene oxide)
Source: Langmuir. 2021 Feb 11;37(7):2362–75. doi: 10.1021/acs.langmuir.0c03302 (PMC8023706; doi:10.1021/acs.langmuir.0c03302)
Supplement: Supplementary file 1 — la0c03302_si_001.pdf [file la0c03302_si_001.pdf]

Supporting Information for **Manipulating Phospholipid Vesicles at the Nanoscale: A Transformation from Unilamellar to Multilamellar by an *n*-Alkyl-poly (ethylene oxide)**

Judith U. De Mel[1*], Sudipta Gupta[1*], Lutz Willner[2], Jürgen Allgaier[2], Laura R. Stingaciu[3], Markus Bleuel[4], and Gerald J. Schneider[1,5*]

[1]Department of Chemistry, Louisiana State University, Baton Rouge, LA 70803, USA

[2]Jülich Center for Neutron Science (JCNS-1) and Institute of Biological Information Processing (IBI-8) Forschungszentrum Jülich GmbH, 52428 Jülich, Germany

[3]Neutron Sciences Directorate, Oak Ridge National Laboratory (ORNL), POB 2008, 1 Bethel Valley Road, TN 37831, Oak Ridge, USA

[4]NIST Center for Neutron Research, National Institute of Standards and Technology, Gaithersburg, Maryland 20899-8562, USA

[5]Department of Physics & Astronomy, Louisiana State University, Baton Rouge, LA 70803, USA

**Correspondence should be addressed to*: ldemel1@lsu.edu, g.sudipta26@gmail.com and gjschneider@lsu.edu**

**Table of Contents**

**Figure S1** Schematic representation of core-shell model Page S2

**Figure S2**. Steps for vesicle preparation Page S2

**Figure S3**. DLS autocorrelation functions and distribution of diffusion coefficients Page S3

**Table S1** Dynamic Light Scattering Results Page S3

**Figure S4**. Additional Cryo-TEM images Page S4

Log-normal distribution Page S4

**Figure S5**. Cryo-TEM images of vesicles with polymer (below and above CMC) Page S5

**Figure S6**. Viscosity data for 5% DOPC dispersed in $D_2O$ Page S5

**Figure S7**. X-ray scattering length density Page S6

**Figure S8** SANS residuals for fitting of Figure 3 Page S6

**Figure S9**. SANS diffraction data Page S7

**Table S2** Calculations lipid polymer interaction ratio Page S7

**Figure S10**. SAXS diffraction data Page S8

**Figure S11**. Caille lamellar structure factor calculated from SAXS Page S8

**Figure S12**. Linear-linear representations of NSE data Page S9

**Figure S13**. Zilman-Granek (ZG) decay rate as a function of momentum transfer, $Q$ Page S10

**Figure S14**. Simulated contrast dependence of MSD Page S10

**Figure S15**. Neutron scattering length density Page S11

## Modified Core-shell Model Structure

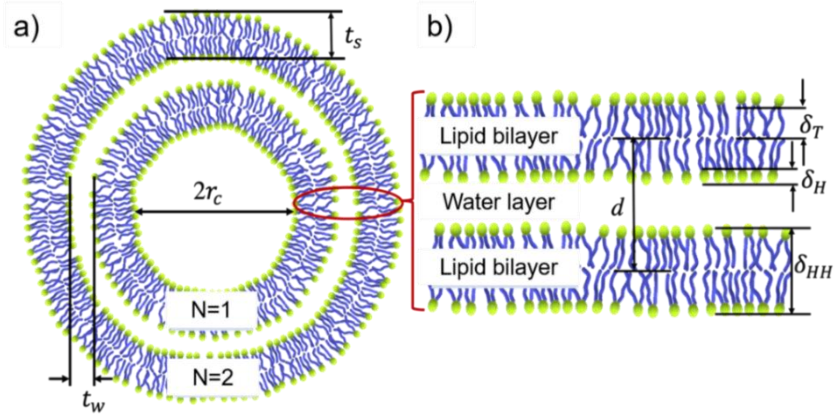


Figure S1. Schematic representation of (a) the multilayer liposome, and (b) lipid multilayers illustrating the number of bilayers, $N$, the radius of the core, $r_c$, the thickness of the individual shells, $t_s$, the thickness of the interleaved solvent layers, $t_w$, the thickness of the lipid head, $\delta_H$, the thickness of the lipid tail region, $\delta_T$ and the lamellar repeat distance, $d$, of bilayers.

## Vesicle Preparation

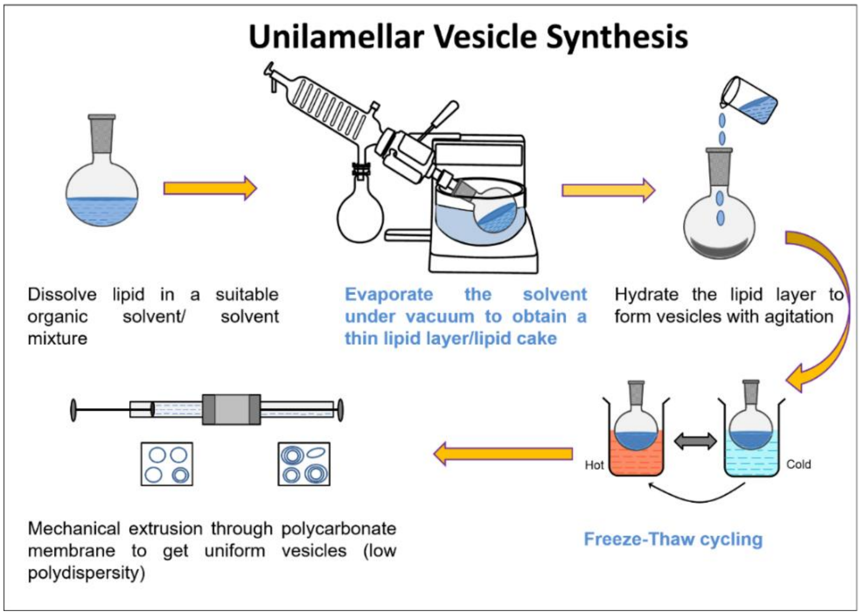


Figure S2. Schematic diagram depicting the steps of vesicle preparation.

## Dynamic Light Scattering (DLS)

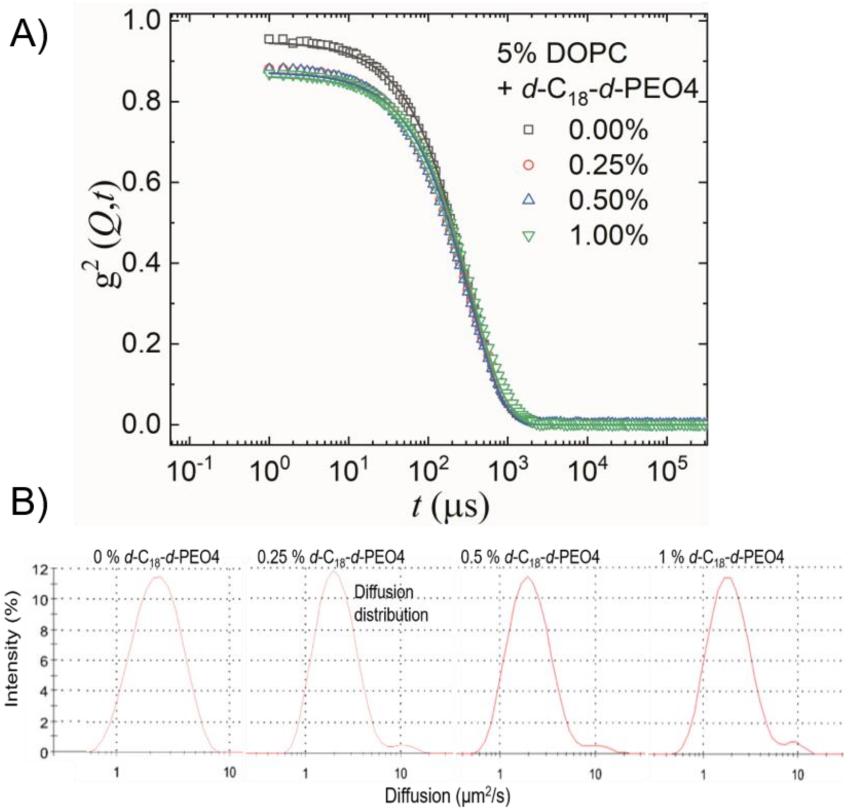


Figure S3. A) Intensity autocorrelation function of DOPC 5wt% vesicles with varying *d*-$C_{18}$-*d*-PEO4 polymer concentrations. The solid lines represent the single diffusion fit (equation 17). B) Distribution of diffusion coefficients for DOPC 5 wt% vesicles with different *d*-$C_{18}$-*d*-PEO4 polymer concentrations (0, 0.25, 0.5, and 1 wt% from left to right). The results are tabulated in Table S1.

Table S1. Dynamic light scattering results of DOPC 5 wt% vesicles with varying *d*-$C_{18}$-*d*-PEO4 polymer concentration. (log-normal size distribution, diffusion coefficient, $D_t$, and hydrodynamic radius, $R_h$, analysis).

| Samples with 5wt% DOPC + *d*-$C_{18}$-*d*-PEO4 | Log-normal size distribution (fit) % | $D_t \times 10^{-12}$ ($m^2s^{-1}$) | Hydrodynamic radius $R_{\mathrm{h}}$ (nm) |
|---|---|---|---|
| 0 wt% | 50.2 ± 0.5 | 2.29 ± 0.02 | 60 ± 2 |
| 0.25 wt% | 48.2 ± 0.5 | 2.17 ± 0.02 | 62 ± 2 |
| 0.5 wt% | 49.8 ± 0.5 | 2.22 ± 0.02 | 61 ± 2 |
| 1 wt% | 49.5 ± 0.5 | 1.98 ± 0.02 | 68 ± 2 |

## Cryo-TEM images and Analysis

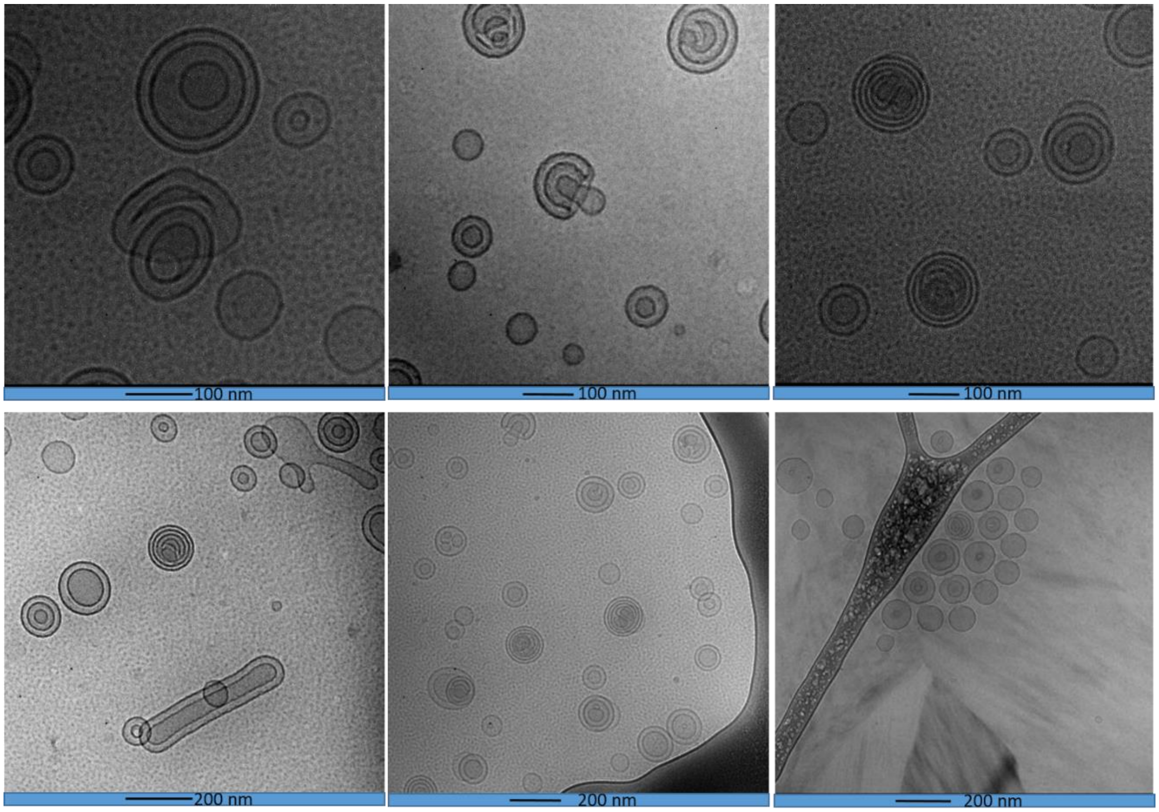


Figure S4. Additional Cryo-TEM images of liposomes in a mixture of 0.25 wt% DOPC and 1 wt% of *d*-$C_{18}$-*d*-PEO4 solutions.

## Log-normal Distribution

For the analysis of the data in the SAXS, SANS, cryo-TEM Data, we used a log-normal distribution given by:

$$s(r) = \frac{1}{\sigma r\sqrt{2\pi}} \exp\left(-\frac{[\ln(r/R_{\text{median}})]^2}{2\sigma^2}\right)$$

where $R_{\text{median}}$ refers to the radius of the particle and, $\sigma$ is the standard deviation representing the polydispersity, $\sigma \times 100$ %.

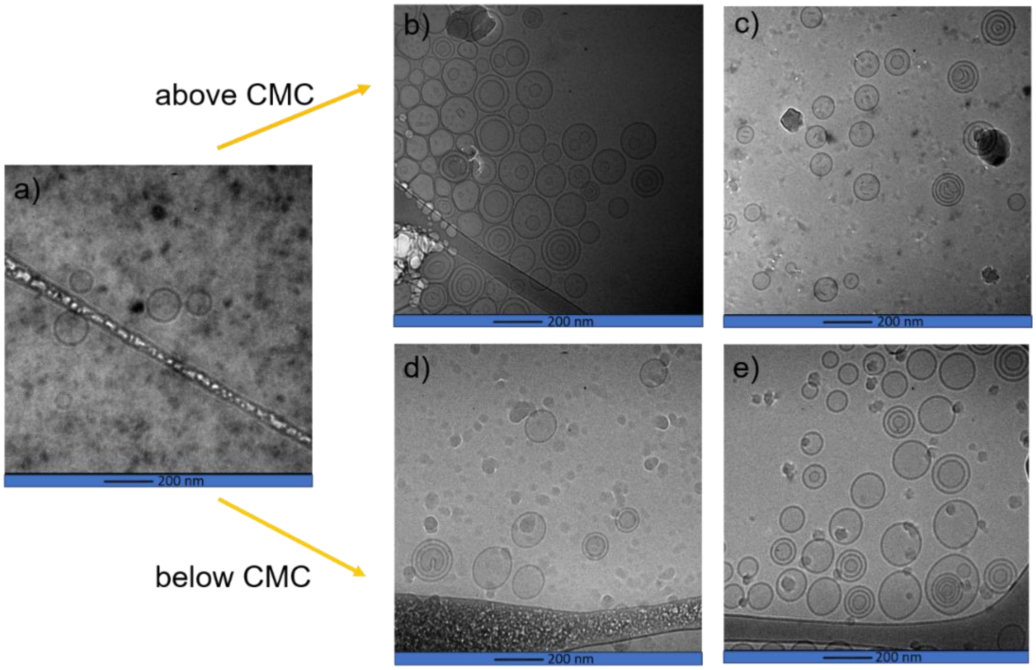


Figure S5. Cryo-TEM images of DOPC / *d*-$C_{18}$-*d*-PEO4 MLVs formed above and below the CMC of polymer a) 0.125 wt% DOPC vesicles b) DOPC+0.25 wt% polymer c) DOPC+0.5 wt% polymer d) DOPC+0.03 wt% polymer e) DOPC+0.06 wt% polymer (*d*-$C_{18}$-*d*-PEO4 CMC = 0.1 wt%) Scale bar = 200 nm

## Viscosity

We used a 20 mm stainless steel cone-plate geometry in Peltier set-up of an AR-2000 rheometer to determine the viscosity at ambient temperature.

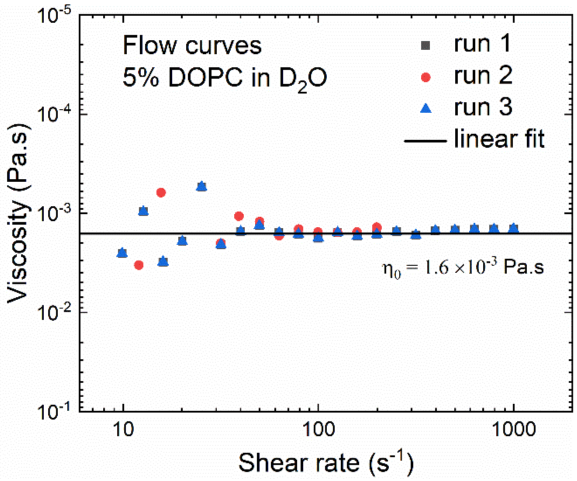


Figure S6. Flow curves illustrating the viscosity as a function of shear rate measured in multiple runs on 5 wt% DOPC in aqueous ($D_2O$) solutions.

## SAXS contrast

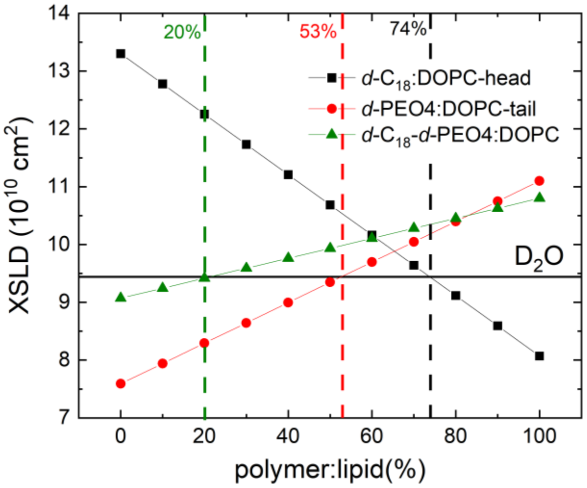


Figure S7. Average X-ray scattering length density (XSLD) as a function of (i) polymer hydrophobic (*d*-$C_{18}$) to lipid (DOPC) head fraction, (ii) polymer hydrophilic (*d*-PEO4) to lipid (DOPC) tail fraction, and (iii) polymer (*d*-$C_{18}$-*d*-PEO4) to lipid (DOPC) fraction. The horizontal line represents the XSLD of $D_2O$. The vertical lines show zero contrast (or contrast match conditions) for hypothetical polymer-to-lipid ratios of 20 % (*d*$C_{18}$-*d*-PEO4/DOPC mixture), 53 % (*d*-PEO4/DOPC-tail), and 74 % (*d*-$C_{18}$/DOPC head) with $D_2O$.

## SANS data

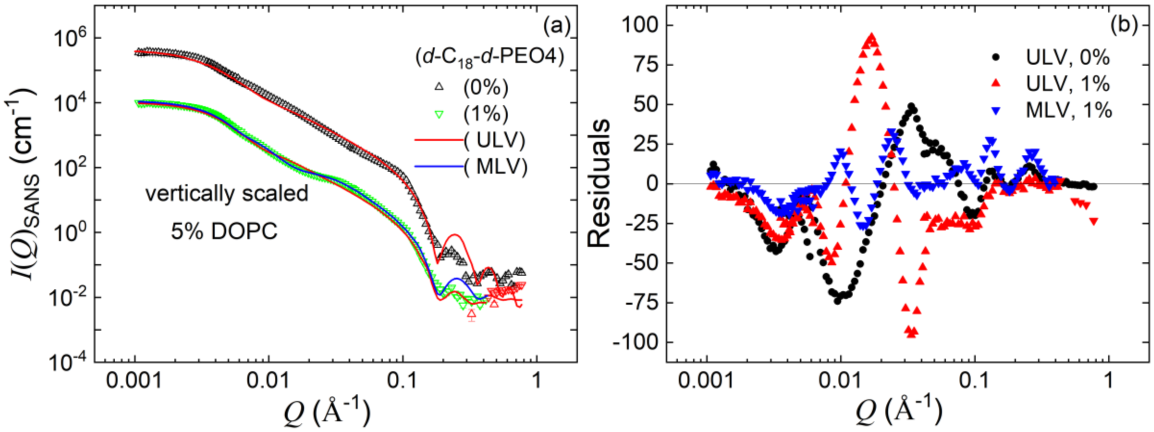


Figure S8. (a) SANS intensity from Figure 3 in the paper. (b) Corresponding residuals normalized by the experimental errors of SANS data. ULV model is compared for with and without polymer (0 wt% and 1 wt%) and MLV model is used for 1 wt.

The residuals for each data point, *i* is given by $R_i = (I(Q)_i - model_i)/error_i$. Here the intensity is plotted in logarithmic scale as shown in figure S8 (a), along with the error bars. The solid lines represent the model.

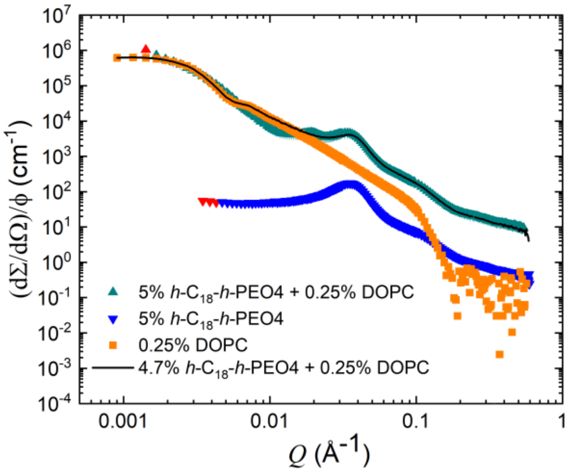


Figure S9. SANS data on $D_2O$ solutions with 0.25 wt% DOPC in $D_2O$ solution, and 5 wt% of *h*-$C_{18}$-*h*-PEO4, and the blend of the two solutions. The black solid line represents the sum of the intensities of the 0.25 wt% DOPC, and 4.7 wt% of *h*-$C_{18}$-*h*-PEO4 solutions

As shown in Figure S9, (5 - 4.7) = 0.3 wt% *h*-$C_{18}$-*h*-PEO4 interacts with 0.25wt% DOPC vesicles, which results in the molecular ratio of DOPC: polymer = 5:1, whereas in figure 3 in the paper when 1 wt% *d*-$C_{18}$-*d*-PEO4 interacts with 5wt% DOPC vesicles, the molecular ratio of DOPC: polymer = 31:1. The calculations are shown below.

**Calculations lipid polymer interaction ratio**

Molecular weights ($M_w$), DOPC: 786 g/mol, *hC$_{18}$-h-PEO4:* 4900 g/mol, *d*-$C_{18}$-*d*-PEO4*:* 4850 g/mol

These calculations for $N = 1$ and 2 assume that only the outer leaflet of the outer bilayer of the vesicle is interacting with the polymers.

Table S2. Calculations for interacting lipid to polymer ratios

| Cases | Interacting molecular ratio in 1 mL solution | |
|---|---|---|
| | DOPC : *h*-$C_{18}$-*h*-PEO4 (0.25wt% : 0.3 wt%) | DOPC : *d*-$C_{18}$-*d*-PEO4 (5wt% : 1 wt%) |
| Total DOPC to polymer | $1.91 \times 10^{18}$: $3.68 \times 10^{17}$= 5: 1 | $3.83 \times 10^{19}$: $1.24 \times 10^{18}$=31: 1 |
| $N = 1$ (2 lipid layers) outer leaflet* | $9.57 \times 10^{17}$: $3.68 \times 10^{17} = 3:1$ | $1.91 \times 10^{19}$: $1.24 \times 10^{18} = 15:1$ |
| $N = 2$ (4 lipid layers) outer leaflet | $6.95 \times 10^{17}$: $3.68 \times 10^{17} = 2:1$ | $1.39 \times 10^{19}$: $1.24 \times 10^{18} = 11:1$ |
| * Assuming both layers have same lipid amount | | |

Therefore, all polymers are inserting to lipid vesicles in the 5wt% DOPC + 1wt% *d*-$C_{18}$-*d*-PEO4 sample since the maximum interacting amount (5.6 wt% *d*-$C_{18}$-*d*-PEO4) has not been reached.

## SAXS data

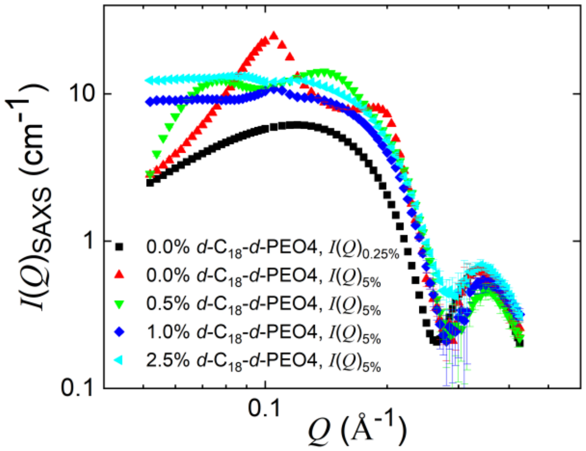


Figure S10. SAXS data for 5 wt% DOPC mixed with 0% to 2.5 wt% of *d*-$C_{18}$-*d*-PEO4 polymer dispersed in $D_2O$. The calculated form factor data for 0.25 wt% DOPC mixed with 0% *d*-$C_{18}$-*d*-PEO4 polymer is included for comparison.

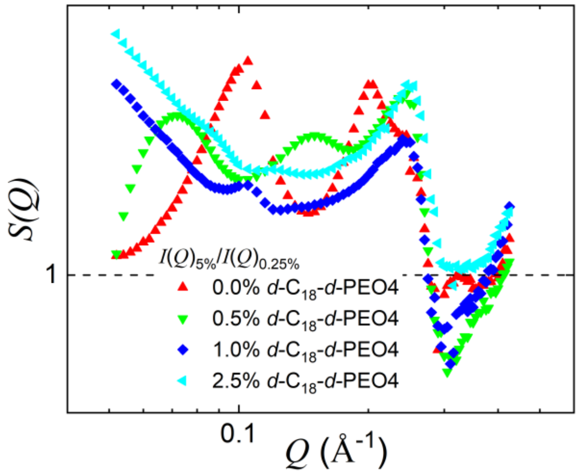


Figure S11. The Caille lamellar structure factor $S(Q)$ calculated from SAXS data for 5 wt% DOPC mixed with 0% to 5 wt% of *d*-$C_{18}$-*d*-PEO4 polymer in $D_2O$. The $S(Q)$ data are calculated by dividing each of the 5 wt% DOPC-polymer data by the 0.25 wt% DOPC data from Figure S10.

NSE data

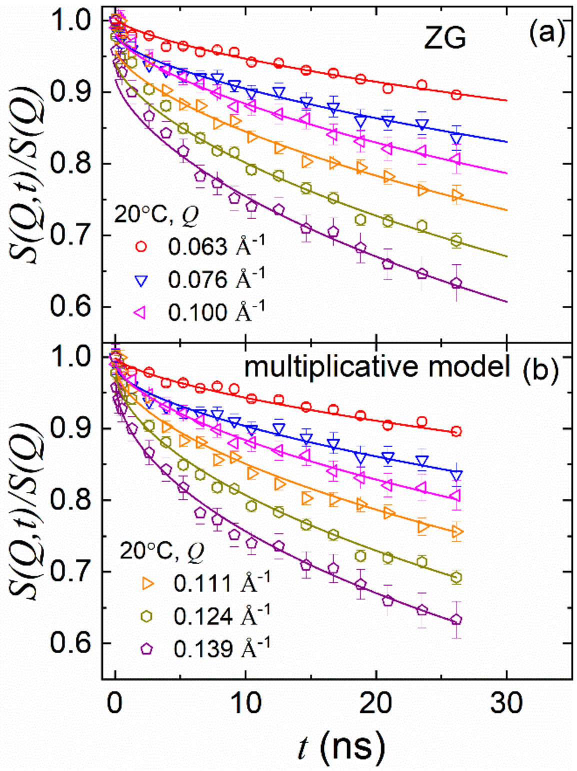


Figure S12. Linear-linear representations of the normalized dynamic structure factor, S(Q,t)/S(Q), as a function of Fourier time, t, for different Q's, on the blend of DOPC and 1 wt% of *d*-$C_{18}$-*d*-PEO4 in $D_2O$ solutions. Temperature is 20°C. The same data sets are analyzed by fits using the (a) Zilman Granek model (equation 13 in the manuscript) and (b) the multiplicative model, which includes translational diffusion of the liposome, Zilman-Granek undulations, and confined motion of the hydrocarbon tails. The error bars representing one standard deviation.

The Zilman-Granek (ZG) decay rate as a function of momentum transfer, $Q$, is presented in Figure S13.

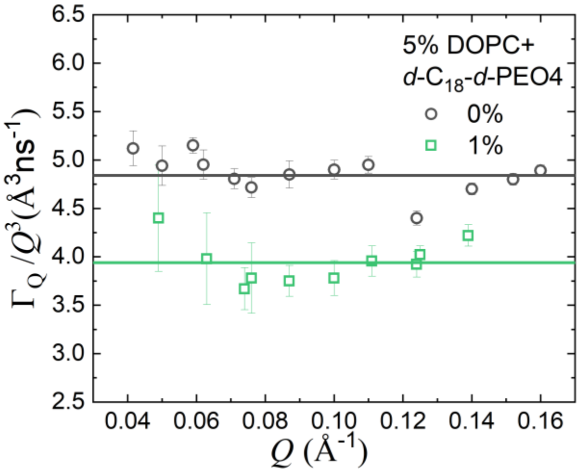


Figure S13. Variation of ZG decay rate, $\Gamma_Q$, as function of $Q$ for different *d*-$C_{18}$-*d*-PEO4 polymer concentration. The ZG decay rate, $\Gamma_Q$, was determined using the multiplicative model. Without the multiplicative model the flat region in the curve is higher by 7.6%.

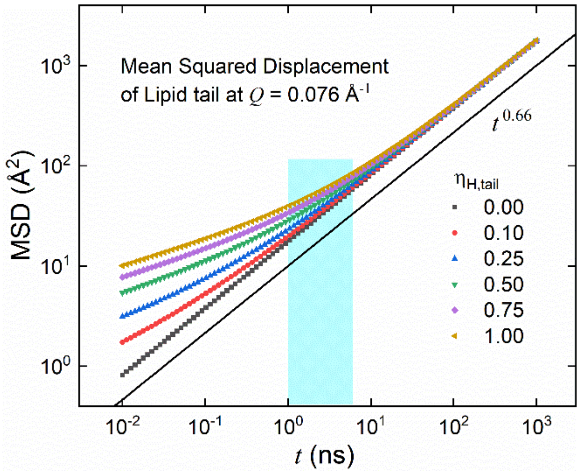


Figure S14. Hypothetical influence of (partial) contrast matching of the lipid tails on the mean square displacement, $\langle \Delta r(t)^2 \rangle$, vs. Fourier time, $t$. From the analysis using the Gaussian assumption at $Q$ = 0.076 Å$^{-1}$. The highlighted region indicates the contrast dependence of the cross-over region from ZG to $t^{0.26}$ power-law dependence of MSD.

## Neutron Contrast

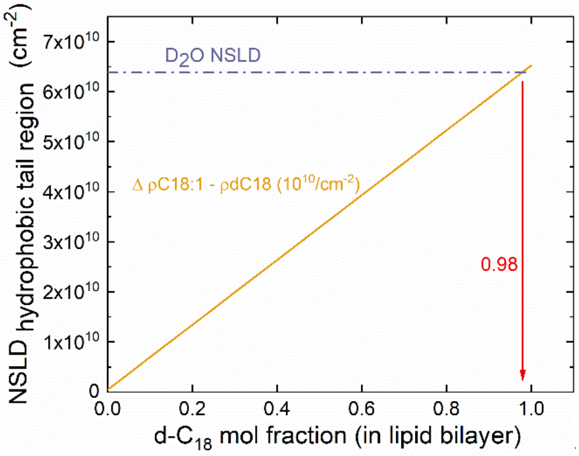


Figure S15. Neutron Scattering Length Density of hydrophobic tail region of bilayer with inserted fraction of *d*-$C_{18}$ chains Horizontal line: NSLD of $D_2O$ for reference Vertical arrow (red): indicates the molar fraction of deuterated tails required to match contrast with $D_2O$ which is 0.98 or 98%. At 5wt% DOPC mixed with 1wt% *d*-$C_{18}$-*d*-PEO4 fraction of deuterated tails may range between 0.02-0.08 which is significantly lower than the required contrast matching conditions for lipid bilayer
